# HEY1-NCOA2 expression modulates chondrogenic differentiation and induces mesenchymal chondrosarcoma in mice

**DOI:** 10.1172/jci.insight.160279

**Published:** 2023-05-22

**Authors:** Miwa Tanaka, Mizuki Homme, Yasuyo Teramura, Kohei Kumegawa, Yukari Yamazaki, Kyoko Yamashita, Motomi Osato, Reo Maruyama, Takuro Nakamura

**Affiliations:** 1 Division of Carcinogenesis, The Cancer Institute, Japanese Foundation for Cancer Research, Tokyo, Japan.; 2 Department of Experimental Pathology, Institute of Medical Science, Tokyo Medical University, Tokyo, Japan.; 3 Project for Cancer Epigenomics, The Cancer Institute, and; 4 Department of Pathology, The Cancer Institute Hospital, Japanese Foundation for Cancer Research, Tokyo, Japan.; 5 Cancer Science Institute of Singapore, National University of Singapore, Singapore.

**Keywords:** Cell Biology, Oncology, Cartilage, Epigenetics, Oncogenes

## Abstract

Mesenchymal chondrosarcoma affects adolescents and young adults, and most cases usually have the *HEY1::NCOA2* fusion gene. However, the functional role of HEY1-NCOA2 in the development and progression of mesenchymal chondrosarcoma remains largely unknown. This study aimed to clarify the functional role of HEY1-NCOA2 in transformation of the cell of origin and induction of typical biphasic morphology of mesenchymal chondrosarcoma. We generated a mouse model for mesenchymal chondrosarcoma by introducing *HEY1-NCOA2* into mouse embryonic superficial zone (eSZ) followed by subcutaneous transplantation into nude mice. *HEY1-NCOA2* expression in eSZ cells successfully induced subcutaneous tumors in 68.9% of recipients, showing biphasic morphologies and expression of *Sox9*, a master regulator of chondrogenic differentiation. ChIP sequencing analyses indicated frequent interaction between HEY1-NCOA2 binding peaks and active enhancers. Runx2, which is important for differentiation and proliferation of the chondrocytic lineage, is invariably expressed in mouse mesenchymal chondrosarcoma, and interaction between HEY1-NCOA2 and Runx2 is observed using NCOA2 C-terminal domains. Although *Runx2* knockout resulted in significant delay in tumor onset, it also induced aggressive growth of immature small round cells. Runx3, which is also expressed in mesenchymal chondrosarcoma and interacts with HEY1-NCOA2, replaced the DNA-binding property of Runx2 only in part. Treatment with the HDAC inhibitor panobinostat suppressed tumor growth both in vitro and in vivo, abrogating expression of genes downstream of HEY1-NCOA2 and Runx2. In conclusion, *HEY1::NCOA2* expression modulates the transcriptional program in chondrogenic differentiation, affecting cartilage-specific transcription factor functions.

## Graphical abstract

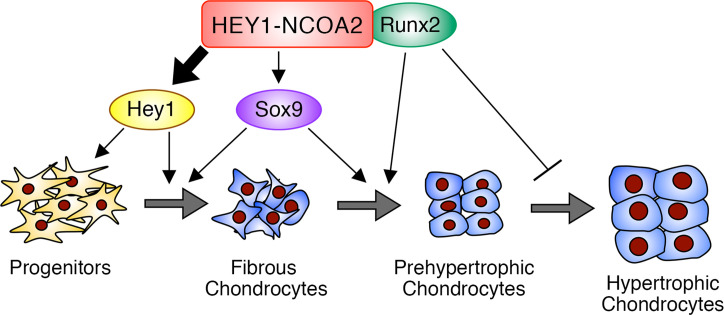

## Introduction

Mesenchymal chondrosarcoma is a rare malignant chondrogenic neoplasm with typical morphological features consisting of primitive mesenchymal cell–like small round to spindle cells and differentiated cartilage (1). The histological and growth characteristics of mesenchymal chondrosarcoma resemble the centripetal growth pattern of embryonic cartilage (2), and the tumor is also characterized by positive expression of Sox9, a master regulator of chondrogenesis (3). The tumors predominantly develop in young adults and adolescents with rather broad range of age distribution (1, 4). The disease involves the bones predominantly, but cases with extraskeletal tissue as a primary site are not rare (5–7). Surgical wide resection of the lesion is recommended as a primary therapy for the localized disease, and systemic cytotoxic chemotherapy is applied for metastatic tumors; however, the overall survival rate remains to be improved (3).

Most cases of mesenchymal chondrosarcoma are associated with a recurrent fusion between *HEY1* and *NCOA2* resulting from intrachromosomal deletion of 8q (4, 8). *HEY1::NCOA2* encodes a chimeric transcription factor composed of a basic helix-loop-helix–type (bHLH-type) DNA-binding domain of HEY1 and 2 transactivation domains derived from NCOA2 (8). The wild-type HEY1 protein functions as a transcriptional repressor bound to E-box located in the promoter or enhancer and a downstream effector of the Notch signaling pathway (9–11). The transcriptional regulation by HEY1 is important for musculoskeletal development, with HEY1 interacting with multiple transcription factors/cofactors such as MyoD and RUNX2 (9, 10). *NCOA2* encodes a transcriptional coactivator consisting of the N-terminal bHLH and C-terminal transactivation domains, the former of which is deleted in the fusion protein (8, 12, 13). NCOA2 interacts with nuclear receptors, recruiting histone methyltransferases (e.g., CARM1 and PRMT1) and major coactivators (e.g., CBP and p300) and resulting in enhanced expression of target genes of nuclear receptors (14–16).

*NCOA2* is involved recurrently in gene fusions associated with soft tissue tumors that include angiofibroma of soft tissue (*AHRR::NCOA2*) and spindle cell/sclerosing rhabdomyosarcoma (*TEAD1::NCOA2* and *VGLL2::NCOA2*) (17–19). It is also found fused to *MYST3* or *ETV6* in acute myeloid leukemia (20–22). Acquisition of the transcriptional activation domains from NCOA2 suggests that HEY1-NCOA2 may act as a transcriptional activator and may upregulate target genes of wild-type HEY1; however, its effect may not be straightforward. MYST3-NCOA2 fusion disrupts the functions of coactivators and transcription factors or modulates chromatin structure, resulting in repression of target genes (23, 24). In contrast, the functional role of HEY1-NCOA2 in the development and progression of mesenchymal chondrosarcoma remains largely unknown (4). An appropriate animal model is therefore needed to clarify the function of HEY1-NCOA2 and the developmental process of the sarcoma.

This study aimed to clarify the functional role of HEY1-NCOA2 in transformation of the cell of origin and induction of typical biphasic morphology of mesenchymal chondrosarcoma. Toward this goal, we generated a mouse model of mesenchymal chondrosarcoma using the same method as that used to generate the Ewing sarcoma mouse model (25).

## Results

### Generation of the mouse model for mesenchymal chondrosarcoma.

**Figure 1:**
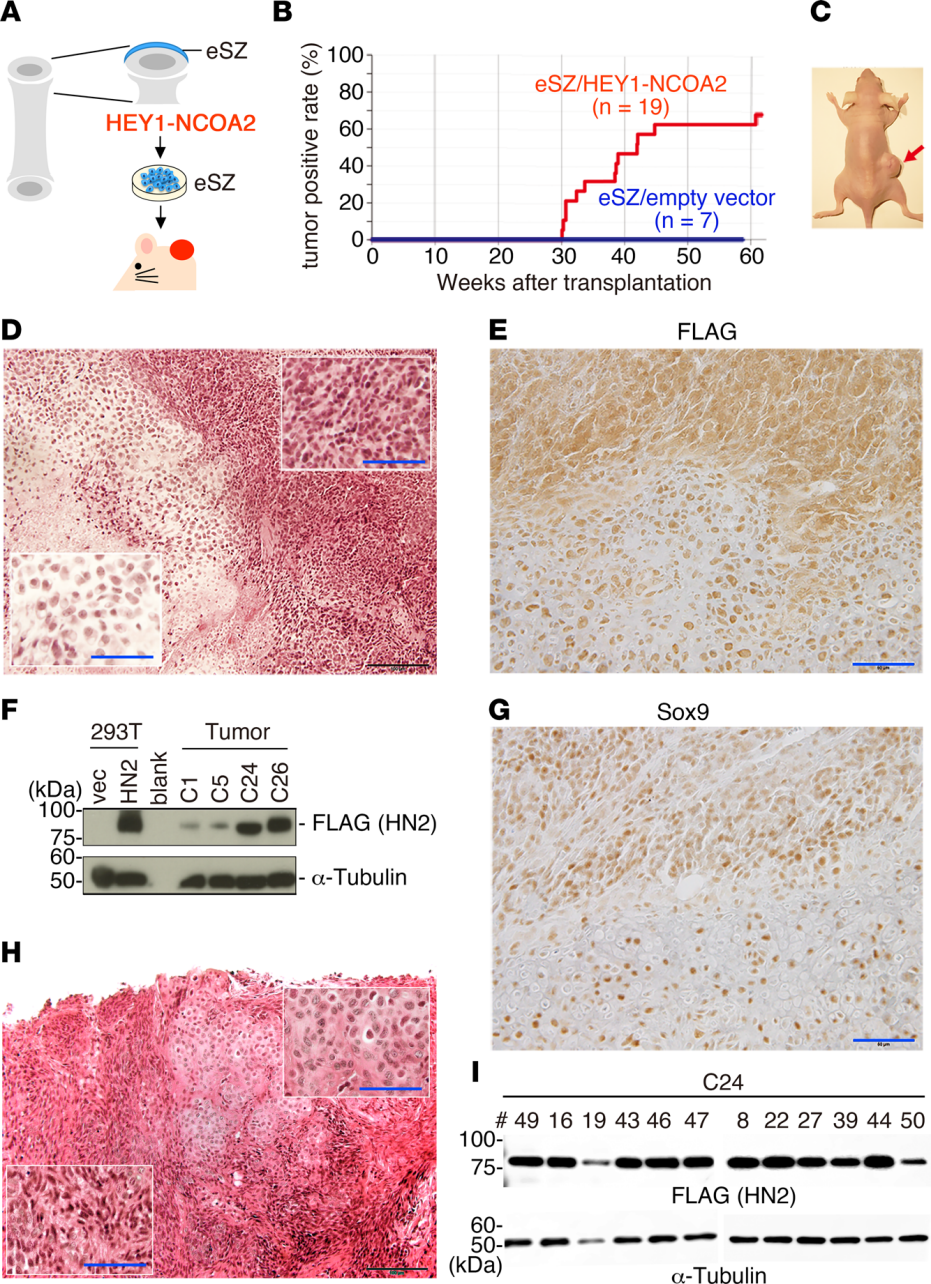
Mouse model for human mesenchymal chondrosarcoma. (**A**) The experimental outline. Embryonic superficial zone (eSZ) is purified by microdissection from femoral and humeral bones. The cells are dissociated and transduced with the *HEY1-NCOA2* retrovirus. After the retroviral infection is completed, the cells are transplanted subcutaneously into nude mice. (**B**) Cumulative incidence (percentage) of mesenchymal chondrosarcoma induced by eSZ cells expressing *HEY1-NCOA2* or with empty vector. (**C**) Tumors (arrow) are observed as subcutaneous masses in recipient nude mice. (**D**) Histology of murine mesenchymal chondrosarcoma. H&E staining shows the biphasic pattern consisting of small round cell proliferation (inset, top right) and mature cartilage (inset, bottom left), which is typical for human mesenchymal chondrosarcoma. Scale bars: 100 μm; 50 μm (insets). (**E**) Immunostaining for anti-FLAG. Scale bar: 50 μm. (**F**) Immunoblotting shows the FLAG-tagged HEY1-NCOA2 protein in tumor tissues. HEK293T cells transfected with HEY1-NCOA2 (HN2) or empty vectors (vec) are used as positive or negative control, respectively (left). (**G**) Immunostaining for anti-Sox9. Scale bar: 50 μm. (**H**) Histology of the single cell–derived tumor. Mature cartilage and immature components are preserved. Scale bars: 100 μm; 50 μm (insets). (**I**) Immunoblotting shows the FLAG-tagged HEY1-NCOA2 protein in 12 single cell–derived clones.

The method to generate the mouse model for mesenchymal chondrosarcoma is outlined in Figure 1A. The FLAG-tagged cDNA encoding a full coding region of human *HEY1::NCOA2* was cloned into a pMYs-IRES-GFP retrovirus vector (Supplemental Figure 1A; supplemental material available online with this article; https://doi.org/10.1172/jci.insight.160279DS1). Embryonic osteochondrogenic progenitor cells purified from the embryonic superficial zone (eSZ) (25) were transduced with the *HEY1-NCOA2* retrovirus. The cells were transplanted subcutaneously into BALB/c nude mice 48 hours after transduction. Subcutaneous mass started to develop 30 weeks after transplantation. In total, 13 of the 19 (68.4%) recipients developed tumors within a 42-week median latency time (Figure 1, B and C). These tumors were serially transplantable to nude mice and did not show distant metastasis spontaneously.

Histological analysis revealed that the tumor consisted of both a mature cartilage-like component and foci of small round to spindle cell proliferation with transient areas between 2 components (Figure 1D), a hallmark of human mesenchymal chondrosarcoma (1). HEY1-NCOA2 expression was confirmed by immunohistochemistry (Figure 1E) and immunoblotting (Figure 1F) using an anti-FLAG antibody. *HEY1::NCOA2* expression was driven by the retroviral promoter/enhancer in our model, whereas it is driven by native regulatory elements of *HEY1*. HEY1-NCOA2 expression was predominantly detected in the immature component of human mesenchymal chondrosarcoma using the antibody recognizing the NCOA2 C-terminal region (Supplemental Figure 1B). The result indicates that expression of HEY1-NCOA2 is different in part between humans and the mouse model. More than 90% of tumor cells showed nuclear expression of Sox9, which is a master regulator of chondrogenic differentiation (3, 26–29) (Figure 1G). This suggested that the mouse mesenchymal chondrosarcoma recapitulates the differentiation process of the chondrogenic lineage and that *HEY1::NCOA2* expression does not significantly inhibit chondrogenic differentiation. *HEY1::NCOA*2 expression in eSZ cells did not affect the expression of *Pthlh*; its expression was enriched in the cell of origin of Ewing sarcoma in our previous model (Supplemental Figure 1C) (25).

Mesenchymal chondrosarcoma consists of hyaline cartilage and immature small tumor cell components. To clarify the single-cell origin of both components, we cloned a series of single cells from mouse tumors and transplanted 12 clones. Biphasic tumors were generated from all clones expressing HEY1-NCOA2 (Figure 1, H and I), indicating that the HEY1-NCOA2–expressing cell possessed the bidirectional differentiation potential.

When mouse embryonic mesenchymal cells purified from limb or trunk soft tissue (*n* = 3 each) were transduced with the *HEY1::NCOA2* retrovirus followed by subcutaneous transplantation into nude mice, mature cartilage tissues developed instead of neoplastic lesions within 40 weeks (Supplemental Figure 1D). This suggested that *HEY1::NCOA* expression may induce chondrogenic differentiation in mesenchymal cells.

### HEY1-NCOA2 modulates expression of cartilage-associated genes.

**Figure 2:**
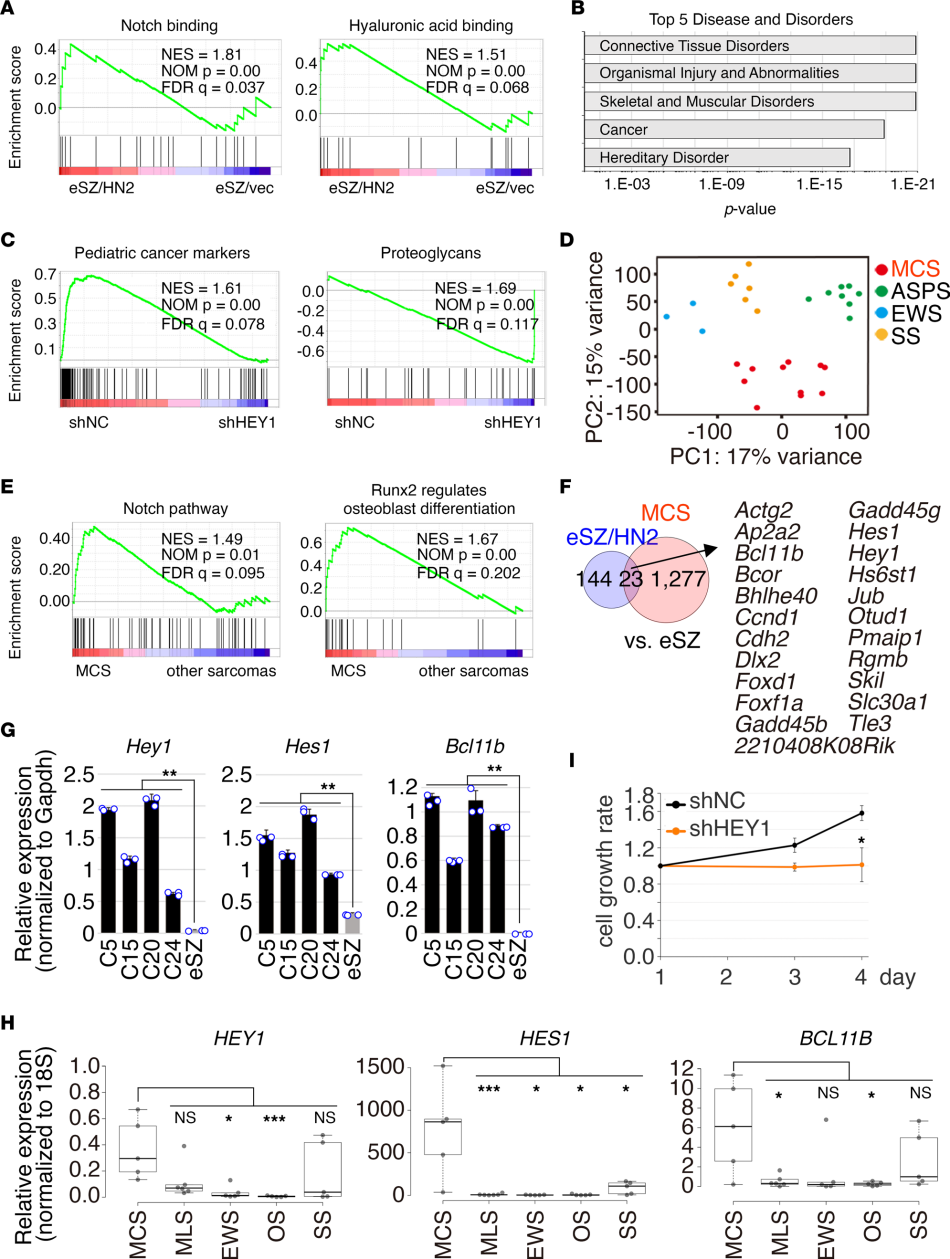
Gene expression profile of murine mesenchymal chondrosarcoma. (**A**) Gene set enrichment analysis (GSEA) shows correlation of Notch signaling and hyaluronic acid binding pathways with genes involved in eSZ cells expressing *HEY1::NCOA2*. (**B**) Systematical analysis of the signaling pathways for 167 upregulated and 48 downregulated genes in mesenchymal chondrosarcoma by the IPA software. The diseases and disorders are listed according to their ranking scores. Blue rectangles indicate each *P* value. *P* values were calculated using Fischer’s exact test. (**C**) GSEA showing inverse and forward correlations between *HEY1-NCOA2* knockdown in mouse mesenchymal chondrosarcoma and pediatric cancer markers and proteoglycans pathways, respectively. The *P* value was computed through the 2-sided permutation test (*n* = 1,000 randomizations) adjusted by the Benjamini-Hochberg procedure. (**D**) Principal component analysis for gene expression profiles of murine mesenchymal chondrosarcoma, Ewing sarcoma, alveolar soft part sarcoma, and synovial sarcoma. (**E**) GSEA shows enrichment of the Notch pathway and the signature for the regulation of osteoblast differentiation by Runx2 in mesenchymal chondrosarcoma. (**F**) Venn diagram showing upregulated genes in *HEY1-NCOA2–*expressing eSZ cells versus eSZ cells containing an empty vector or mesenchymal chondrosarcoma versus eSZ cells containing an empty vector. (**G**) qRT-PCR shows upregulated expression of *Hey1, Hes1,* and *Bcl11b* in mesenchymal chondrosarcoma cells. (**H**) Growth suppression of mesenchymal chondrosarcoma cells C24 by shRNA-mediated gene silencing of *HEY1-NCOA2* in vitro. (**I**) qRT-PCR shows increased expression of *HEY1, HES1*, and *BCL11B* in human mesenchymal chondrosarcoma tissues. ASPS, alveolar soft part sarcoma; MCS, mesenchymal chondrosarcoma (*n* = 5); MLS, myxoid liposarcoma (*n* = 6); EWS, Ewing sarcoma (*n* = 6); OS, osteosarcoma (*n* = 5); SS, synovial sarcoma (*n* = 5). Statistical analyses in **G** and **H** were performed by 1-way ANOVA and in **I** were performed by 2-sided Student’s *t* test. *P < 0.05, **P < 0.01, ***P < 0.001.

Gene expression profiles were compared between eSZ cells with and without introduction of *HEY1::NCOA2* 48 hours after transduction. A total of 167 and 48 genes were upregulated or downregulated by more than 1.5-fold in eSZ cells with *HEY1::NCOA2* expression, respectively (Supplemental Table 1). Gene set enrichment analysis (GSEA) revealed that the expression of genes associated with Notch signaling and metabolism related to chondrogenesis was positively correlated with *HEY1::NCOA2* expression (Figure 2A and Supplemental Figure 2A). The cartilage development pathway was also positively correlated with eSZ cells expressing HEY1-NCOA2 compared with embryonic limb mesenchymal cells (Supplemental Figure 2A). Ingenuity Pathway Analysis (IPA) revealed gene pathways involved in connective tissue disorders or skeletal and muscular disorders as highly relevant pathways (Figure 2B). When *HEY1::NCOA2* was knocked down using human *HEY1*-specific shRNA sequences, genetic pathways associated with pediatric cancer and proteoglycan synthesis were affected (Figure 2C, Supplemental Figure 2B, and Supplemental Table 2).

In contrast, when gene expression profiles were compared between sarcoma cells and eSZ cells, expression of chondrocyte differentiation and cartilage-associated metabolic pathway genes was inversely correlated with sarcoma cells (Supplemental Figure 2C). Quantitative reverse transcription PCR (qRT-PCR) confirmed downregulated expression of *Comp* and *Matn3*, genes expressed in differentiated chondrocytes (30, 31) (Supplemental Figure 2D). Collectively, these data indicate that *HEY1::NCOA2* expression in chondrogenic progenitors ameliorates the chondrogenic differentiation program and that the effect of HEY1-NCOA2 is cellular-context dependent. The gene expression profile of mouse mesenchymal chondrosarcoma was then compared with that of other murine sarcomas (Ewing sarcoma, synovial sarcoma, and alveolar soft part sarcoma) (25, 32, 33).

Principal component analysis (PCA) exhibited that mesenchymal chondrosarcoma possessed a distinct feature from other sarcomas (Figure 2D). Moreover, *HEY1-NCOA2* expression was associated with pathways involved in Notch, osteoblast differentiation by Runx2, chondroitin sulfate/dermatan sulfate metabolism, and the BMP pathway (Figure 2E and Supplemental Figure 2E). These data indicate that HEY1-NCOA2 could act as an oncogene when it is properly expressed in the cell of origin and that HEY1-NCOA2 does not simply function as suppressor of chondrogenic differentiation. Comparison of gene expression profiles between tumor cells and chondrogenic progenitors introduced with *HEY1::NCOA2* identified 23 common upregulated genes (Figure 2F). qRT-PCR analysis confirmed upregulation of *Hey1,* *Hes1,* and *Bcl11b* (Figure 2G), suggesting that *HEY1::NCOA2* positively regulates endogenous *Hey1* expression and modulates the Notch/Hey1 axis. Expression of *HEY1, HES1*, and *BCL11B* was also increased in human mesenchymal chondrosarcoma compared with other sarcomas such as myxoid liposarcoma, Ewing sarcoma, osteosarcoma, and synovial sarcoma (Figure 2H). In addition, gene expression profiles of eSZ cells expressing HEY1-NCOA2 or mouse mesenchymal chondrosarcoma cells were compared with that of mature chondrocyte (34). The analysis identified 2,182 common upregulated genes, and GSEA identified enrichment of gene pathways of mesenchymal cell proliferation, cell cycle, and stem cell development (Supplemental Figure 2, F and G, and Supplemental Table 3), suggesting dynamic modulation of cell proliferation–associated genes by HEY1-NCOA2 expression. Finally, shRNA-mediated gene silencing of *HEY1::NCOA2* abrogated growth of mesenchymal chondrosarcoma cells in vitro (Figure 2I), indicating that the survival and proliferation of mesenchymal chondrosarcoma are dependent on *HEY1::NCOA2* expression.

### Single-cell analysis of mouse mesenchymal chondrosarcoma demonstrates incomplete chondrogenic differentiation.

**Figure 3:**
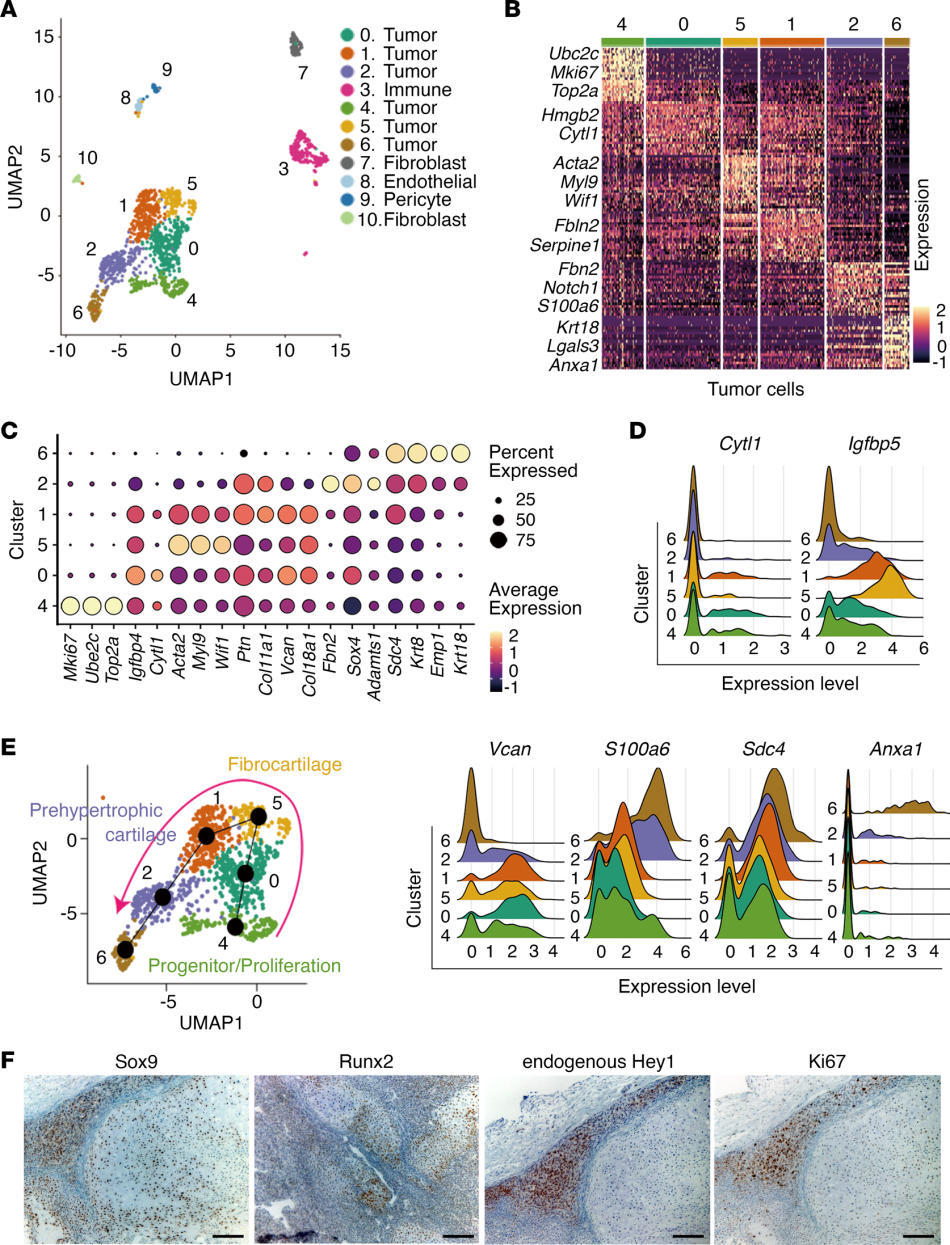
Gene expression network during chondrogenic differentiation of mesenchymal chondrosarcoma revealed by single-cell RNA sequencing and immunostaining. (**A**) scRNA-Seq followed by PCA-guided uniform manifold and projection (UMAP) identifies neoplastic and nonneoplastic cellular fractions. Clusters are divided into 0–10 as indicated on the right. (**B**) Heatmap revealing 120 differentially expressed genes for each cluster defined in **A**. (**C**) Dot plot analysis using 19 chondrogenesis-related genes that represent each tumor cluster. (**D**) Differential expression of *Cytl1, Igfbp5, Vcan, S100a6, Sdc4*, and *Anxa1* was further demonstrated by ridge plot. (**E**) Pseudotime trajectory analysis showing a differentiation pathway from progenitor/proliferation cluster (see 4) to more differentiation stages via fibrocartilage (see 5) and prehypertrophic cartilage (see 2). (**F**) Immunohistochemical analysis demonstrates distinct expression patterns with partial overlapping of Sox9, Runx2, endogenous Hey1, and Ki67 in mouse mesenchymal chondrosarcoma. Scale bar: 100 μm.

To analyze the differentiation steps in mesenchymal chondrosarcoma, single-cell RNA sequencing (scRNA-Seq) was performed on a single case of mouse mesenchymal chondrosarcoma. In total, 3,489 cells were identified and used for subsequent analysis. Using PCA-guided uniform manifold and projection (UMAP), 10 transcriptionally distinct cell populations consisting of both neoplastic and nonneoplastic components were identified (Figure 3A). Among these 10 populations, 6 clusters containing 2,930 cells were determined as neoplastic cells that express multiple cartilage-related genes (Figure 3B). Dot plot, ridge plot, and pseudotime trajectory analyses demonstrated differentiation stage-specific gene expression and incomplete chondrogenic differentiation process from progenitors (cluster 4, characterized by *Mki67, Ube2c,* and *Top2a*) to prehypertrophic cartilage-like component (cluster 2, *Ptn, Col11a1, Vcan*, and *Col18a1*) (Figure 3, C–E). Distribution of the fibrocartilage component between progenitors and prehypertrophic cartilage is consistent with previous studies using scRNA-Seq on human cartilage (35, 36). Expression of *Krt8, Krt18,* and *Lgals3,* which are notochord markers (37), was observed in cluster 6, suggesting that dysregulated differentiation might also occur in mesenchymal chondrosarcoma, unlike normal chondrocytic differentiation. Distribution of other important gene expressions such as *Runx2, Hey1, Pthlh, Sox9, Col2a1*, or *Ihh* failed to show very informative data, partly due to relative low expression levels of these genes (Supplemental Figure 3A). Immunohistochemical analysis of human mesenchymal chondrosarcoma cases also showed positive expressions of CK18 and GAL3, gene products of KRT18 and LGALS3, respectively (Supplemental Figure 3A). Immunohistochemistry for Ki67 both in mouse and human mesenchymal chondrosarcoma showed that progenitor/proliferation cluster 4 was mainly composed of small round cell fractions (Figure 3F and Supplemental Figure 3B). Chondrocyte-associated transcription factors Sox9 and Runx2 were positive for both mature and immature components, whereas endogenous Hey1 was predominantly expressed in immature round cell fraction (Figure 3F and Supplemental Figure 3B).

### HEY1-NCOA2 is frequently associated with active enhancers including super enhancers.

**Figure 4:**
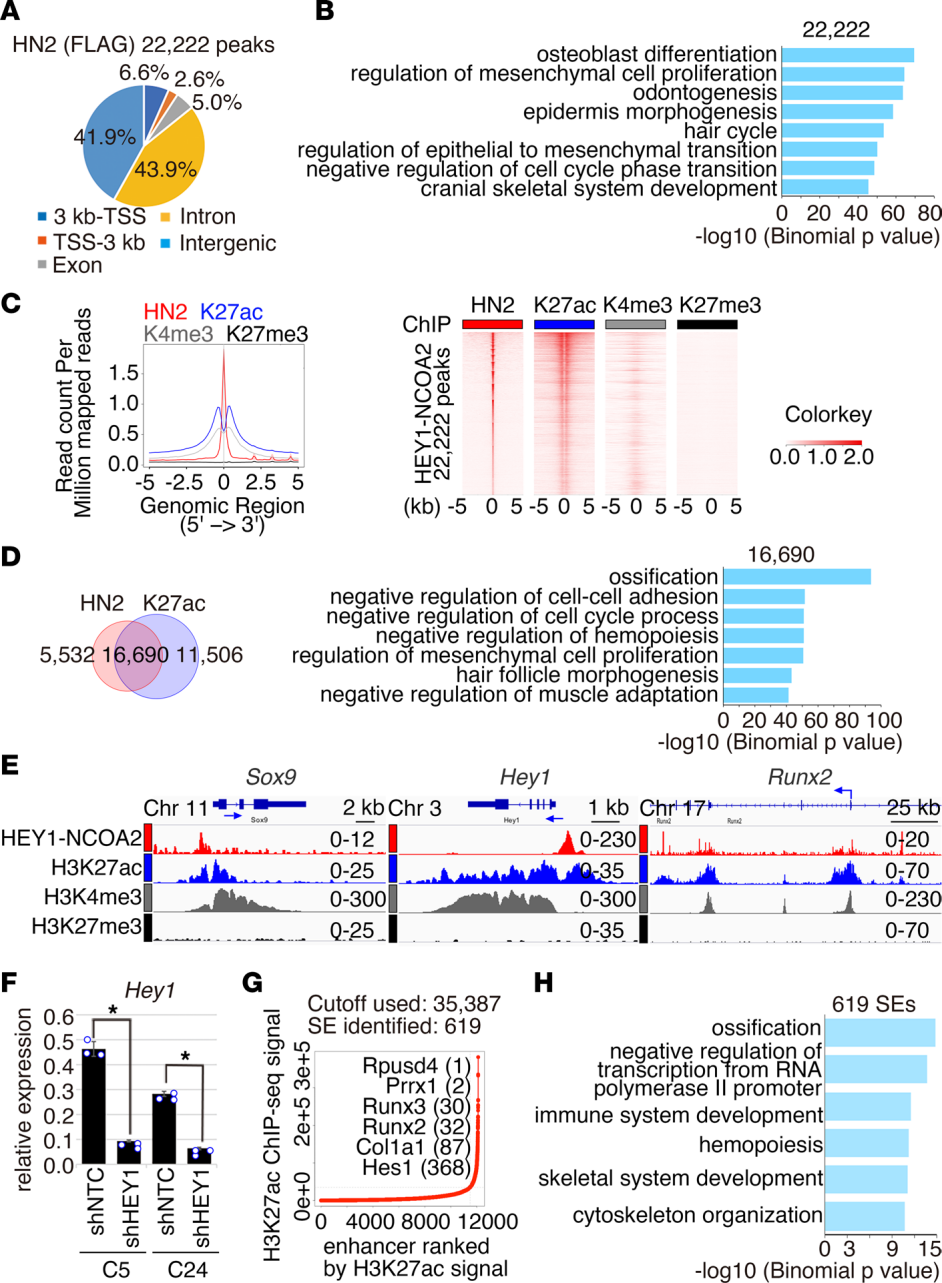
HEY1-NCOA2 binding sites in mesenchymal chondrosarcoma. (**A**) Global distribution of HEY1-NCOA2 binding peaks in the mouse mesenchymal chondrosarcoma cell C24. (**B**) The GREAT gene ontology analysis identifies important genetic pathways. Blue rectangles indicate each *P* value. (**C**) Composite plots (left) and heatmap (right) of HEY1-NCOA2, H3K27ac, H3K4me3, and H3K27me3 signals centered on HEY1-NCOA2 binding peaks in mouse MCS. (**D**) Venn diagram showing overlapping between HEY1-NCOA2 (HN2) and H3K27ac binding peaks. Enrichment of gene ontology biological process for overlapping peaks is indicated in right. (**E**) ChIP-Seq occupancy profiles for *Sox9*, endogenous *Hey1,* and *Runx2* loci. Arrows indicate transcriptional orientation and the transcriptional start site for *Runx2*. (**F**) Quantitative RT-PCR showing downregulation of *Hey1* by *HEY1::NCOA2* silencing. The statistical analysis was performed by Student’s *t* test. *P < 0.05. (**G**) Enhancers are ranked by increasing H3K27ac ChIP-Seq signals in mesenchymal chondrosarcoma cells. Using the ROSE algorithm, 619 enhancers are defined as super enhancers (SEs). (**H**) Gene ontology analysis of 619 SEs. *P* values in **B**, **D**, and **H** were calculated using a binominal test.

To further clarify the role of HEY1-NCOA2 in gene expression, ChIP assay with sequencing (ChIP-Seq) was performed to investigate HEY1-NCOA2 binding and histone modifications in mouse mesenchymal chondrosarcoma cells. The HEY1-NCOA2 binding peaks were mainly distributed in intronic areas (43.9%) and intergenic regions (41.9%) (Figure 4A, Supplemental Figure 4A, and Supplemental Table 4). The Genomic Regions Enrichment of Annotations Tool (GREAT) gene ontology analysis (http://great.stanford.edu/public/html/) for HEY1-NCOA2 binding peaks identified osteogenic pathways and mesenchymal cell proliferation (Figure 4B). Moreover, 75% of HEY1-NCOA2 binding peaks overlapped with histone H3K27ac peaks, suggesting that gene regulation on osteogenic pathways and mesenchymal cell proliferation by HEY1-NCOA2 were achieved via the enhancer function (Figure 4, C–E, and Supplemental Figure 4B).

DNA binding of HEY1-NCOA2 was observed at the promoter region of endogenous *Hey1*, and its expression was positively regulated by HEY1-NCOA2, as was confirmed by knockdown of *HEY1::NCOA2* (Figure 4, E and F). As previously described, nuclear expression of wild-type Hey1 was confirmed in mouse mesenchymal chondrosarcoma (Figure 3F). These data suggest that HEY1-NCOA2 may upregulate wild-type *Hey1* in a limited fraction of sarcoma cells. HEY1-NCOA2 binding accompanied by H3K27ac accumulation was also observed at *Sox9, Runx2, Runx3,* and *Hes1* loci, suggesting the possible regulatory role of HEY1-NCOA2 in expression of these genes. Super enhancers (SEs) in tumor cells were then examined because they define cell identity and are often associated with genes involved in cancer development (38, 39). The ROSE analysis (http://younglab.wi.mit.edu/super_enhancer_code.html) identified 619 SEs, including *Hes1,* *Runx2,* and *Runx3,* and pathway analysis for SE-identified ossification and skeletal system development pathways (Figure 4, G and H, and Supplemental Table 5). Collectively, these findings support that HEY1-NCOA2 upregulates osteochondrogenic differentiation via active enhancers and, at least in part, SEs.

### HEY1-NCOA2 is associated with RUNX2 in mesenchymal chondrosarcoma.

**Figure 5:**
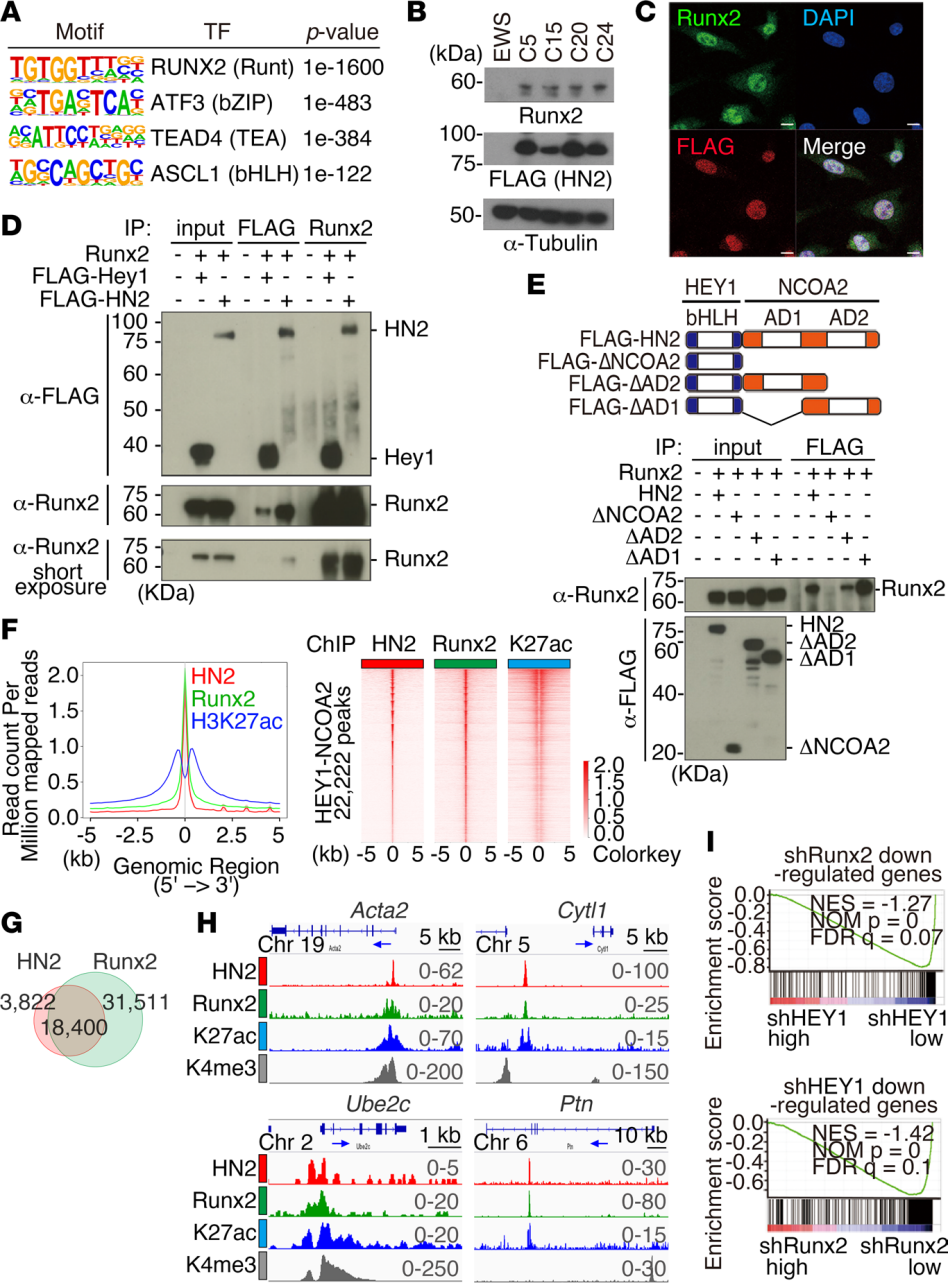
Association between HEY1-NCOA2 and Runx2 in DNA binding. (**A**) HOMER motif analysis showing enrichment of the RUNX2 motif in HEY1-NCOA2 binding peaks detected in mouse mesenchymal chondrosarcoma cell C24. *P* values were calculated using Fischer’s exact test. (**B**) Expression of the Runx2 protein in mesenchymal chondrosarcoma cells (C5, C15, C20, and C24). Ewing sarcoma cells (EWS) are used as a negative control. (**C**) Immunofluorescent assessment of the expression of FLAG-tagged HEY1-NCOA2 and endogenous Runx2 in C24 cells. Scale bar: 10 μm. (**D**) HEK293T cells are transiently transfected with FLAG-tagged HEY1-NCOA2, FLAG-tagged wild-type Hey1 and Runx2. The cell lysates are immunoprecipitated with anti-FLAG or anti-Runx2 antibodies and immunoblotted with anti-FLAG or anti-Runx2 antibodies. (**E**) The schematic diagram of HEY1-NCOA2 deletion mutants (top). bHLH, basic helix-loop-helix domain; AD1 and AD2, activation domain 1 and 2. Coimmunoprecipitation assays using the above constructs and the full-length Runx2 are performed similarly to **D**. (**F**) Composite plots (left) and heatmap (right) of HEY1-NCOA2, Runx2, and H3K27ac ChIP-Seq data sets centered on HEY1-NCOA2 binding peaks. (**G**) Venn diagram showing overlapping between HEY1-NCOA2 (HN2) and Runx2 binding peaks. (**H**) ChIP-Seq occupancy profiles for *Acta2, Cytl1, Ube2c*, and *Ptn* loci. Arrows indicate transcriptional orientation. (**I**) GSEA showing correlation in gene expression profiles between silencing of *HEY1-NCOA2* and *Runx2*.

The HOMER motif analysis of HEY1-NCOA2 binding peaks identified a RUNX consensus sequence as the most frequent binding peak (Figure 5A and Supplemental Figure 5A). Meanwhile, E-box (CATGTG), a putative HEY1-binding motif, had lower significance in the HEY1-NCOA2 binding peaks (Figure 5A and Supplemental Figure 5A). The considerable expression of 3 *Runx* genes (Supplemental Table 1) and the presence of the SE at the *Runx2* and *Runx3* genomic loci (Figure 4G) suggest that HEY1-NCOA2 might interact with a RUNX family protein, especially RUNX2. RUNX2 plays a key role in osteochondrogenic differentiation (40–43), and it is commonly expressed in mouse mesenchymal chondrosarcoma. In the current study, the level of RUNX2 expression varied among the individual tumors (Figure 5B).

Immunofluorescence of mouse mesenchymal chondrosarcoma cells showed nuclear colocalization between HEY1-NCOA2 and endogenous Runx2 (Figure 5C). This nuclear colocalization was also confirmed by transient expression of HEY1-NCOA2 and Runx2 in HEK293T cells (Supplemental Figure 5B). Coimmunoprecipitation experiments revealed the interaction between HEY1-NCOA2 and Runx2 (Figure 5, D and E). A previous study showed that wild-type Hey1 interacts with RUNX2 using both bHLH and orange domains in chondrocytes (44). In the current study, despite the lack of the orange domain of HEY1, HEY1-NCOA2 interacted with RUNX2 using the C-terminal transactivation domain (AD2) with minor contributions of AD1 (Figure 5E).

ChIP-Seq showed frequent association of HEY1-NCOA2 and Runx2 binding signals both in histone H3K27ac-rich active enhancer and nonenhancer regions, with 82% of HEY1-NCOA2 peaks associated with Runx2 peaks (Figure 5, F and G). Genes involved in osteoblast differentiation and mesenchymal cell proliferation were enriched in the overlapping peaks. Furthermore, peaks were observed near genes important for chondrocyte functions and proliferation, including *Acta2, Cytl1, Ube2c,* and *Ptn* (Figure 5H and Supplemental Figure 5C), suggesting the importance of HEY1-NCOA2 and Runx2 association in these functional pathways. Moreover, 481 of 619 SEs (78%) included both HEY1-NCOA2 and Runx2 peaks (Supplemental Figure 5D). The association of DNA binding peaks and protein interaction between HEY1-NCOA2 and Runx2 suggests the presence of downstream target genes co-regulated by both transcription factors.

This was supported by GSEA showing that the sets of downregulated genes upon silencing of *Runx2* were significantly enriched in those also downregulated upon silencing of *HEY1::NCOA2* (Figure 5I and Supplemental Figure 5E). Retrovirus integration sites in 12 tumors were investigated to identify possible cooperating genes with *HEY1::NCOA2* in tumorigenesis, and 3 common integration sites, namely, *Runx2, Palld,* and *Wwc2,* were identified (Supplemental Figure 5F). No significant up- or downregulation in *Runx2* expression by retroviral integrations was observed (Supplemental Figure 5G). Analysis using a larger cohort may clarify the role of the integration at the *Runx2* locus. Collectively, these results underscore the role of Runx2 and its interaction with HEY1-NCOA2 in disease phenotypes of mesenchymal chondrosarcoma.

### Runx2 modifies gene regulation and differentiation programs in mesenchymal chondrosarcoma.

**Figure 6:**
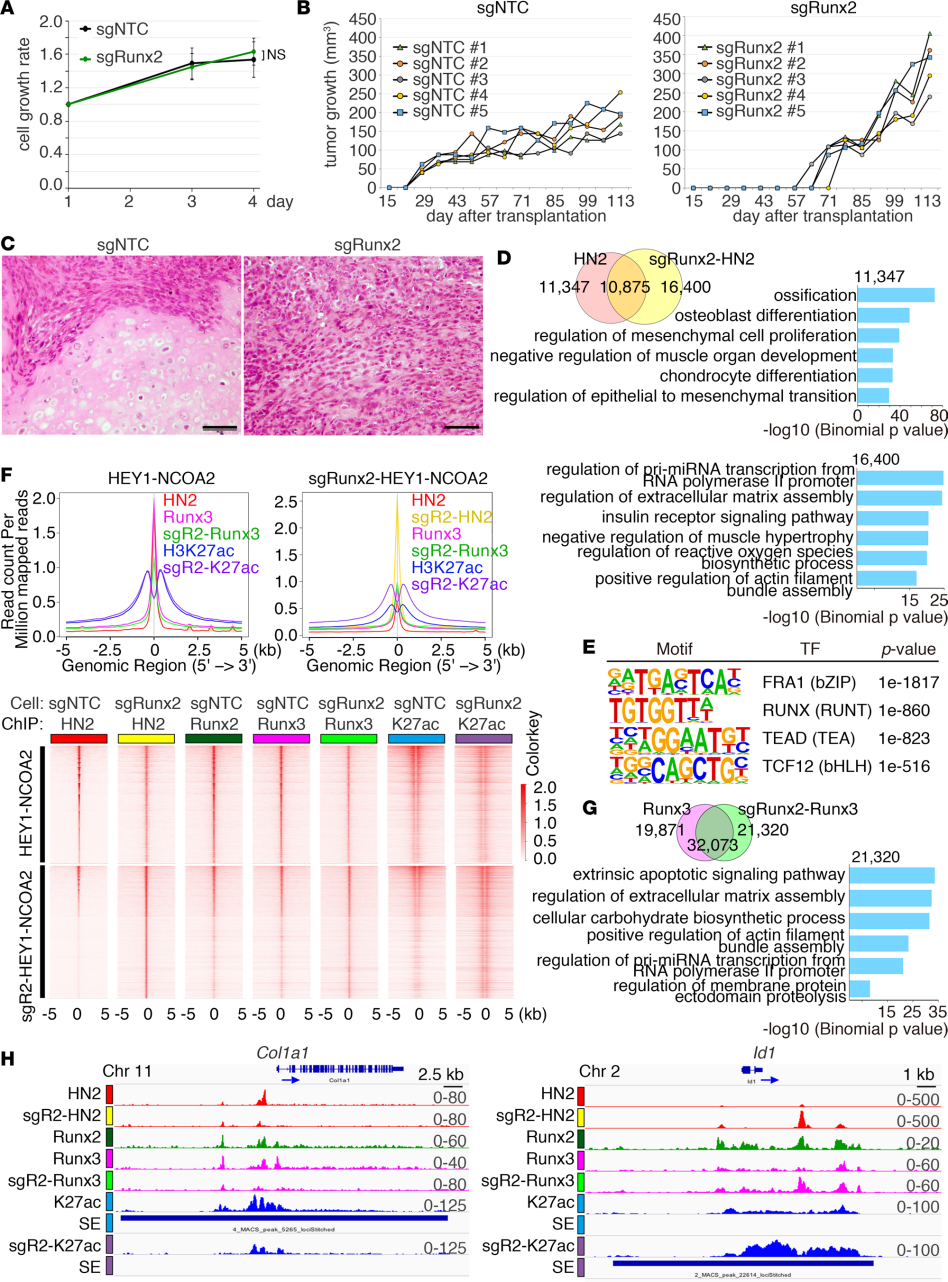
Transcriptional regulation by HEY1-NCOA2 and Runx2 collaboration. (**A**) CRISPR/Cas9-mediated knockout of *Runx2* does not suppress cell growth of mouse mesenchymal chondrosarcoma cells C24. The statistical analysis was performed by Student’s *t* test. (**B**) In vivo tumor growth of mouse mesenchymal chondrosarcoma C24 cells by *Runx2* knockout. Volumes of each tumor are indicated. (**C**) Histology of representative tumors with control (sgNTC) and *Runx2* knockout (sgRunx2) in **B**. Scale bar: 50 μm. (**D**) Venn diagram showing the overlapped distribution of HEY1-NCOA2 (FLAG) with or without *Runx2* knockout (top). Enrichment of gene pathways for 11,347 peaks specific to *Runx2*-positive and 16,400 peaks specific to *Runx2*-negative conditions is indicated (bottom). (**E**) HOMER motif analysis showing partial reduction of RUNX motif enrichment in HEY1-NCOA2 binding peaks upon *Runx2* knockout. *P* values were calculated using Fischer’s exact test. (**F**) Composite plots (top) and heatmap (bottom) of HEY1-NCOA2 (HN2) Runx2, Runx3, and H3K27ac signals centered on HEY1-NCOA2 binding peaks with or without *Runx2* knockout. Plots are centered on HEY1-NCOA2 peaks with *Runx2* expression (top) or knockout (bottom). (**G**) Venn diagram showing the overlapped distribution of Runx3 peaks with or without *Runx2* knockout (left). Enrichment of gene pathways for 21,320 Runx3 peaks appeared by *Runx2* knockout (right). (**H**) Modification of ChIP-Seq occupancy profiles for HEY1-NCOA2, Runx2, Runx3, and H3K27ac at the *Col1a1* and *Id1* loci by *Runx2* knockout. The arrows indicate transcriptional orientation. SE, super enhancers. *P* values in **D** and **G** were calculated using a binominal test.

Despite the significant interaction between HEY1-NCOA2 and Runx2, CRISPR/Cas9-mediated homozygous deletion of *Runx2* failed to show growth suppression of tumor cells in vitro (Figure 6A and Supplemental Figure 6, A and B). In contrast, *Runx2* knockout induced delay in tumor onset but more rapid growth after onset in vivo (Figure 6B). Interestingly, the mature cartilage component disappeared in *Runx2*-deleted tumors and monotonous proliferation of the immature round cell fraction became predominant (Figure 6C). To confirm whether DNA binding of HEY1-NCOA2 was modified by Runx2 knockout, distribution of the binding peaks was investigated by ChIP-Seq.

The DNA-binding profile of HEY1-NCOA2 was substantially modified by *Runx2* knockout, with loss in 51% of original peaks and occurrence of 16,400 unique peaks (Figure 6D and Supplemental Figure 6C). The pathway analysis revealed that the 11,347 peaks lost in *Runx2* knockout were associated with osteoblast differentiation and mesenchymal cell proliferation programs. They were also associated with acquisition of peaks associated with insulin response and extracellular matrix functions. Although motif analysis of HEY1-NCOA2 binding peaks demonstrated decreased frequency of the RUNX motif, it remained significantly more frequent than the bHLH motif (Figure 6E). The result suggests that other Runx family proteins may replace, in part, Runx2 DNA binding.

Therefore, we next examined DNA binding of Runx3, which is another Runx member associated with cartilage and bone development (45). The majority of Runx3 binding sites were associated with those of Runx2 and HEY1-NCOA2, and 38% of these Runx3 peaks disappeared by *Runx2* knockout (Figure 6, F and G). Conversely, unique peaks or peaks with increased signals of Runx3 were observed, and these peaks were frequently associated with those of HEY1-NCOA2 original and new peaks (Figure 6F). The SE signature was significantly modified by *Runx2* knockout (Supplemental Figure 6, E and F). *Col1a1* is a target gene of HEY1-NCOA2 and Runx2, and HEY1-NCOA2 binding and SE activity were lost by Runx2 deletion (Figure 6H). *Id1* is a target gene involved in cell cycle progression (46) that became what we believe to be a novel target for both HEY1-NCOA2 and Runx3 by Runx2 loss. A potentially novel SE also emerged (Figure 6H).

### Runx3 interacts with HEY1-NCOA2, and Runx3 knockout does not affect sarcoma phenotypes in vivo.

**Figure 7:**
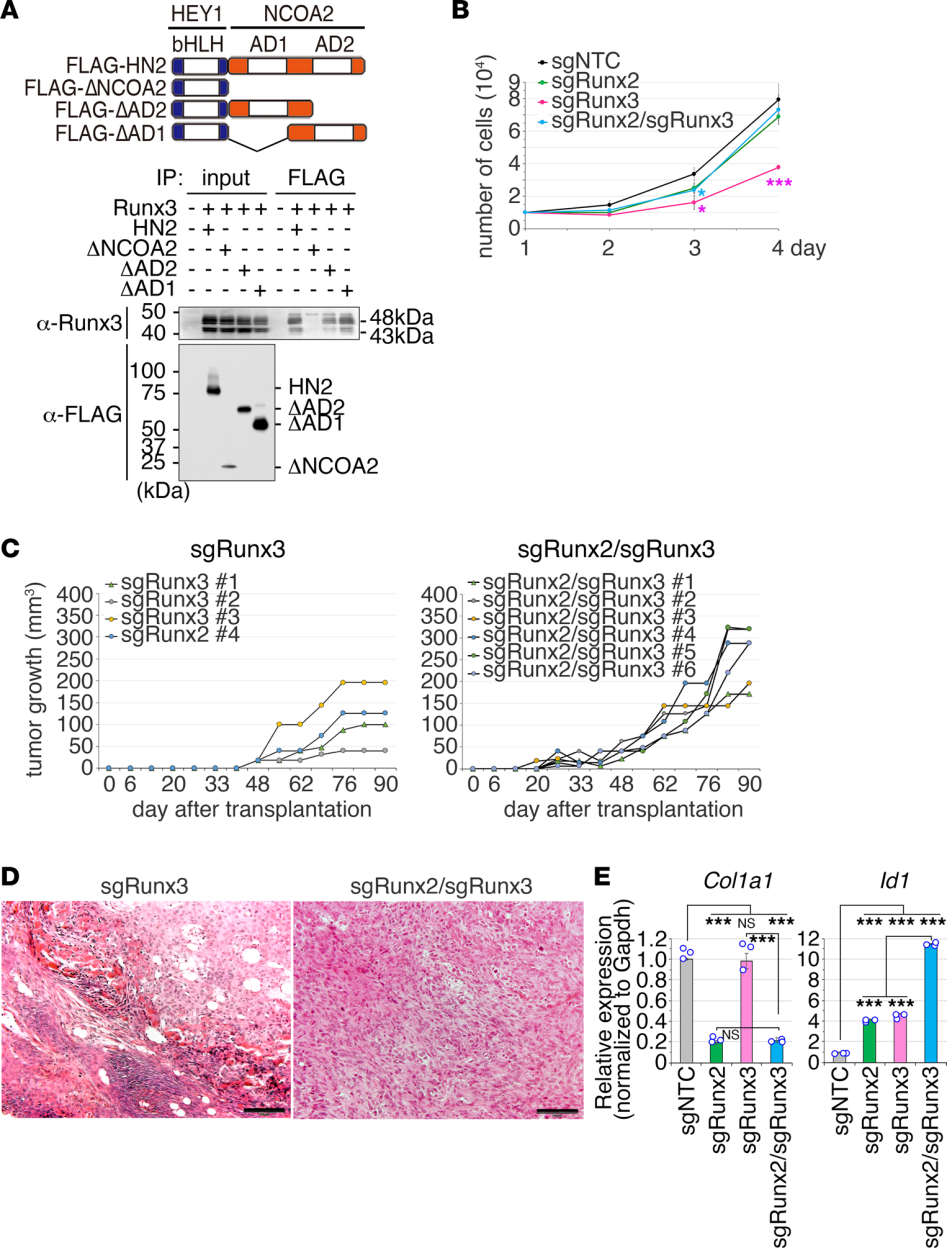
The role of Runx3 in growth and differentiation of mesenchymal chondrosarcoma. (**A**) Coimmunoprecipitation assays for HEY1-NCOA2 and Runx3. HEK293T cells are transiently transfected with FLAG-tagged HEY1-NCOA2, its deletion mutants used in Figure 5E, and Runx3. The schematic diagram of HEY1-NCOA2 deletion mutants (top). The cell lysates were immunoprecipitated with an anti-FLAG antibody and immunoblotted with anti-FLAG or anti-Runx3 antibodies. (**B**) *Runx3* knockout suppresses cell growth of mouse MCS cells C24 but not *Runx2*-knockout cells. (**C**) In vivo tumor growth of mouse MCS C24 cells by *Runx3* knockout (sgRunx3) and *Runx2/Runx3* double knockout (sgRunx2/sgRunx3). Volumes of each tumor are indicated. (**D**) Histology of representative tumors with *Runx3* knockout and *Runx2/Runx3* double knockout in **C**. Scale bar: 50 μm. (**E**) Expression of *Col1a1* and *Id1* in *Runx2, Runx3* knockout and double knockout examined by qRT-PCR. Statistical analysis in **B** was performed by 2-sided Student’s *t* test and in **E** was performed by 1-way ANOVA. *P < 0.05, ***P < 0.001.

Frequent association in DNA binding between HEY1-NCOA2 and Runx3 suggests the possible interaction of both proteins. Coimmunoprecipitation experiments revealed the interaction between HEY1-NCOA2 and Runx3 using C-terminal activation domains derived from NCOA2 (Figure 7A). Homozygous deletion of *Runx3* exhibited growth suppression of wild-type mesenchymal chondrosarcoma cells but not *Runx2*-knockout cells in vitro (Figure 7B and Supplemental Figure 7), whereas growth property of sarcoma in vivo was not affected significantly by *Runx3* knockout both in wild-type and *Runx2*-knockout sarcoma cells (Figure 7C). Moreover, biphasic morphology consisting of differentiated cartilage and immature cell components were well preserved by *Runx3* knockout (Figure 7D). Although expression of *Col1a1* was not altered by *Runx3* knockout, expression of *Id1* was upregulated by *Runx2/Runx3* double knockout (Figure 7E), suggesting that HEY1-NCOA2 might modulate *Id1* transcription in the absence of Runx family proteins. Taken together, the role of Runx3 in the differentiation of mesenchymal sarcoma was limited compared with that of Runx2, although *Runx3* knockout affected cell growth in vitro.

### HDAC inhibitor suppresses mesenchymal chondrosarcoma growth.

**Figure 8:**
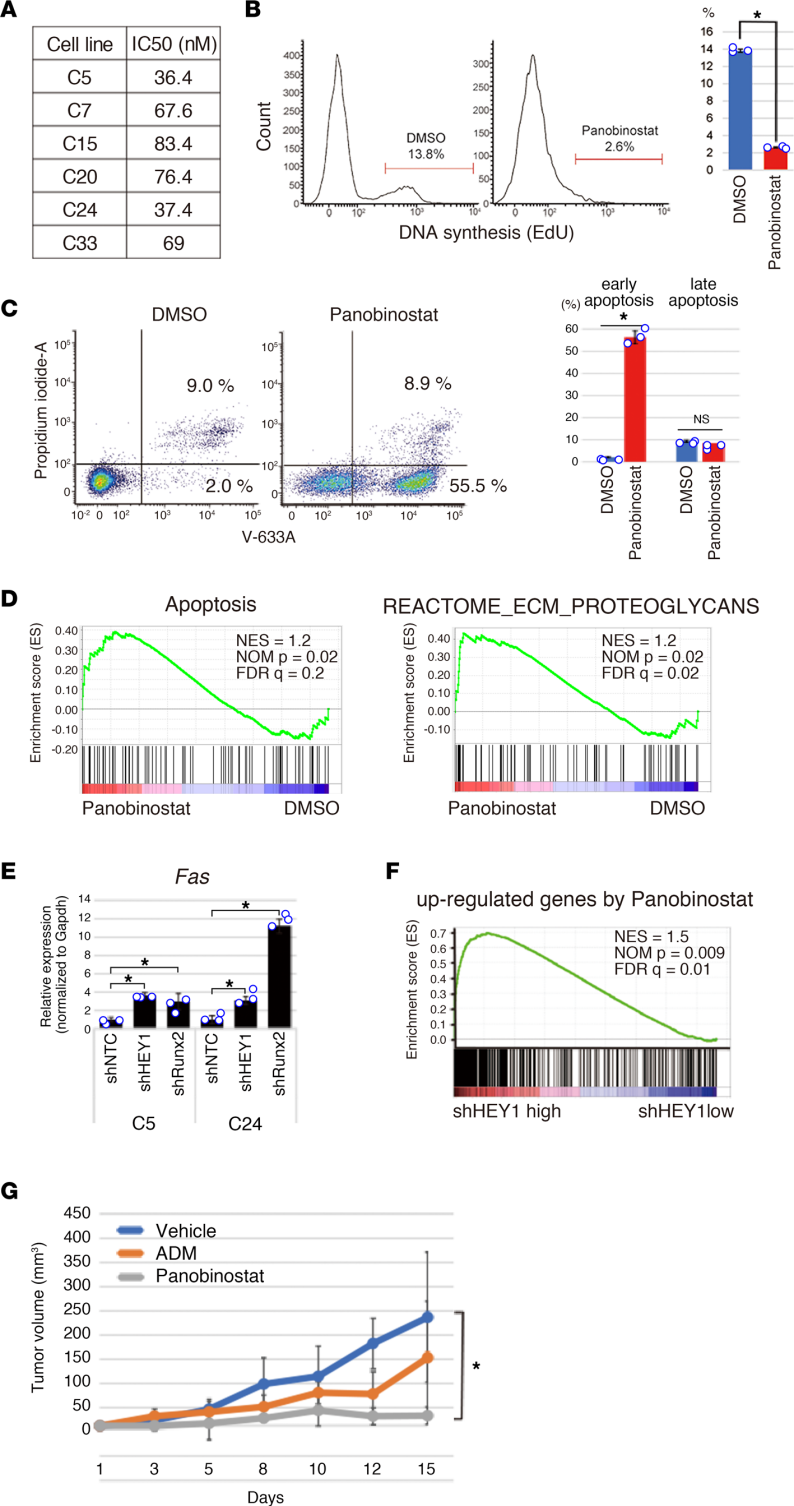
Panobinostat treatment inhibits the growth of mesenchymal chondrosarcoma. (**A**) Half-maximal inhibitory concentration (IC50) of panobinostat for 6 different mouse mesenchymal chondrosarcoma cell lines. (**B**) Suppression of EdU incorporation in C24 cells by panobinostat treatment (10 nM). The representative example is shown on the left, and the average EdU incorporation with SEM is shown on the right. (**C**) Detection of apoptosis induced by panobinostat treatment for 24 hours. Annexin V staining shows a significant increase in both early and late apoptotic cells, as evidenced by flow cytometry (left) and quantified by bar graphs (right). (**D**) GSEA shows enrichment of the apoptosis and extracellular matrix/proteoglycans genetic pathways by panobinostat treatment. (**E**) Upregulation of *Fas* expression by silencing of *HEY1::NCOA2* and *Runx2* in C5 and C24 cells (left). (**F**) GSEA shows correlation in gene expression profiles between panobinostat treatment and silencing of *HEY1::NCOA2*. (**G**) Growth suppression of mesenchymal chondrosarcoma by panobinostat in vivo. The recipient animals (*n* = 5 in each group) are treated with adriamycin (ADM) or panobinostat, and the tumor sizes are measured. Statistical analyses in **B**, **C**, and **G** were performed by 2-sided Student’s *t* test and in **E** was performed by 1-way ANOVA. *P < 0.05.

To explore therapeutic approaches for mesenchymal chondrosarcoma, our model was used as a preclinical platform to evaluate drug effects for mesenchymal chondrosarcoma. Although drug-sensitivity screening using a library of 334 compounds (Screening Committee of Anticancer Drugs [SCADS]) (47) failed to identify highly effective chemicals (Supplemental Table 6), modulations of histone deacetylase functions have been emphasized in fusion gene-positive sarcomas as well as the Runx2 pathway in chondrosarcoma (48, 49). We therefore tested HDAC inhibitors and identified panobinostat (Selleckchem) as an effective growth inhibitor. When mesenchymal chondrosarcoma was treated with panobinostat, significant growth suppression was observed in vitro with rather small deviation of half-maximal concentration (IC_50_) (Figure 8A). Panobinostat treatment also induced suppression of DNA synthesis and promotion of apoptosis (Figure 8, B and C).

The expression of apoptosis-related genes and genes associated with extracellular matrix/proteoglycans was significantly enriched (Figure 8D), indicating that panobinostat treatment affects cell survival and differentiation. In this context, *Fas* upregulation was induced by silencing of HEY1-NCOA2 and Runx2 with DNA binding of both transcription factors (Figure 8E). In addition, there was significant correlation between panobinostat-induced modification of gene expression and gene silencing of *HEY1-NCOA2* (Figure 8F). The significant growth suppression by panobinostat was also confirmed in vivo and the tumor inhibitory effect of panobinostat was greater than that of adriamycin (Figure 8G). Collectively, these results indicate that HDAC inhibitors are promising therapeutic reagents for mesenchymal chondrosarcoma.

## Discussion

This study demonstrated that human *HEY1::NCOA2* expression in embryonic chondrogenic progenitors successfully developed mesenchymal chondrosarcoma. The tumor showed typical biphasic pattern consisting of small round cells and mature cartilage. These results indicate that the embryonic chondrogenic progenitor is, at least in part, the cell of origin of mesenchymal chondrosarcoma. The long latency period and incomplete penetrance of tumor induction, however, suggest that cooperative factors and additional enrichment of the cell-of-origin fraction might be required. Interestingly, *HEY1::NCOA2* expression could induce metaplastic cartilage but not neoplastic transformation when it is expressed in embryonic mesenchymal cells of soft tissue, suggesting that HEY1-NCOA2 might activate the differentiation of chondrocytes in mesenchymal progenitors. In support of this idea, *Sox9* expression was maintained upon *HEY1::NCOA2* introduction throughout the carcinogenic process of mesenchymal chondrosarcoma, as *Sox9* is expressed at high level in eSZ cells. In addition, *Sox9* transcription might be regulated by HEY1-NCOA2 maintenance of its SE. These findings provide what we believe to be a novel insight into the biology of mesenchymal chondrosarcoma and the function of HEY1-NCOA2.

Our previous study identified eSZ cells as the cell of origin of Ewing sarcoma (25), and the present study indicates overlapping of the cell of origin between Ewing sarcoma and mesenchymal chondrosarcoma. Moreover, wild-type *Hey1* upregulation was regulated by HEY1-NCOA2, which is important for Notch-induced oncogenic processes (50). Unlike Sox9 expression, endogenous Hey1 is mainly present in the small round cell component, suggesting the different role in chondrogenesis between 2 transcription factors. Single-cell RNA-Seq analysis identified the differential expression of key gene sets associated with chondrogenic differentiation that recapitulates embryonic chondrogenic development in the model.

Several studies have emphasized the important role of Runx2 in chondrogenic development (40–43). A previous study reported that Runx2 interacts with wild-type Hey1 using its orange domain and that Hey1 represses Runx2-regulated transcription of target genes (44). The present study found that the C-terminal region of NCOA2, including activation domains, compensates for the lack of the orange domain, affecting the Runx2 function in the chondrogenic differentiation program. Frequent overlapping of DNA-binding and physical interaction between HEY1-NCOA2 and Runx2 indicates that there is a co-regulatory mechanism of their target genes.

Homozygous deletion of *Runx2* induced dynamic alteration of tumor component and modification in the distribution of HEY1-NCOA2 DNA-binding peaks. This suggests that Runx2 is at least required for chondrogenic differentiation at later stages, which may be responsible for the characteristic biphasic pattern of mesenchymal chondrosarcoma. ChIP-Seq analysis revealed that Runx3, another member of Runx family transcription factors, also participated in the majority of HEY1-NCOA2 and Runx2 binding sites; however, Runx3 did not completely compensate Runx2 function in chondrogenic differentiation. Instead, *Runx2* deletion induced SE remodeling and drastic changes in target gene expression.

Treatment of mesenchymal chondrosarcoma with the HDAC inhibitor panobinostat led to growth suppression both in vitro and in vivo. The result is consistent with the previous results on the effective growth inhibition in fusion gene-positive sarcomas such as Ewing sarcoma, alveolar rhabdomyosarcoma, and synovial sarcoma (51–53). Correlated modulation of gene expression between panobinostat treatment and silencing of HEY1-NCOA2 or Runx2 indicated that downregulation of target genes by HEY1-NCOA2 and Runx2 cooperation, at least in part, is important for tumor cell maintenance. Given that wild-type Hey1 functions as a transcriptional repressor recruiting HDAC (50) and is upregulated as a target of HEY1-NCOA2, panobinostat may target Hey1-regulated histone deacetylation.

Panobinostat treatment induced apoptosis with upregulation of *Fas* in mouse mesenchymal chondrosarcoma, highlighting the importance of the Fas-mediated cell death pathway, which was reported in the treatment of osteosarcoma by the HDAC inhibitor, SNDX-275 (54). The MOZ-TIF2 (MYST3-NCOA2) fusion protein induces impairment of the coactivator CBP and represses the expression of multiple downstream genes in a promoter context-dependent manner (23, 24), suggesting that HEY1-NCOA2 is involved in chromatin remodeling and explaining panobinostat rescue of the expression of the differentiation-associated genes. In agreement with this, a series of Hey1 target genes such as Col2a1, which is downstream of the Notch signaling pathway (55), were activated by HEY1-NCOA2.

**Figure 9:**
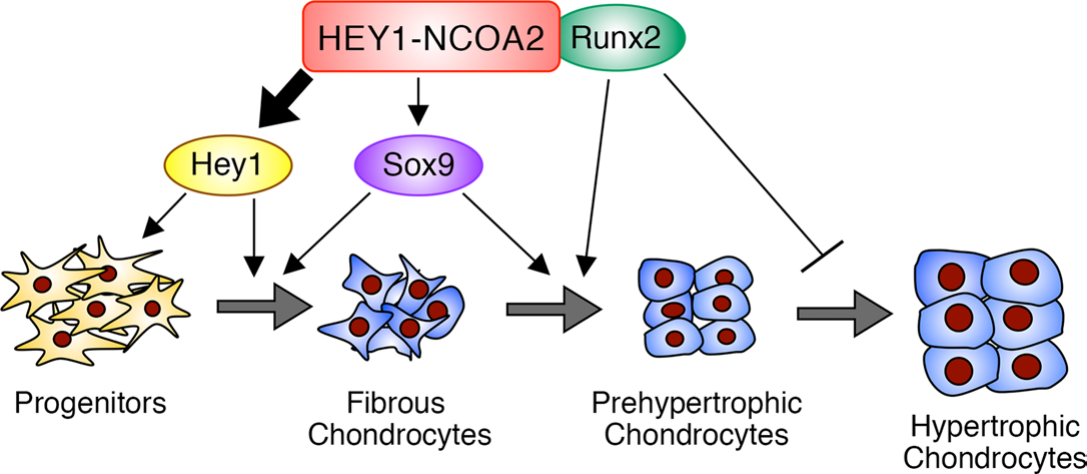
Model of HEY1-NCOA2 in mesenchymal chondrosarcoma development. HEY1-NCOA2 upregulates endogenous Hey1 to promote proliferation of progenitor cells and supports Sox9 to maintain the differentiation program. Concurrently, HEY1-NCOA2 interacts with Runx2 to induce prehypertrophic chondrocytes, but this interaction also suppresses terminal differentiation.

HEY1-NCOA2 disrupts fine tuning of differentiation-related genes by Sox9, Runx2, and Hey1 via DNA binding and interaction with Runx2. Runx2 is required for chondrogenic differentiation at later stages, maintaining the characteristic morphologies of mesenchymal chondrosarcoma. Loss of Runx2 results in immature round cell proliferation that might be promoted by Hey1 (Figure 9). In addition, terminal differentiation cartilage is abrogated, as was suggested by abnormal expression of notochord markers at the end of fate in pseudotime trajectory analysis, which might be caused by HEY1-NCOA2–regulated abnormal gene expression.

This study has some limitations on the functional relationship between HEY1-NCOA2 and Runx2. Although *Runx2* knockout induced more aggressive phenotypes, the function of Runx2 is not a simple suppressor in tumorigenesis, given that *Runx2* knockout did not alter cell proliferation in vitro. Interaction between Runx2 and HEY1-NCOA2 suggests that cooperative and/or competitive regulation of their target genes may be responsible for incomplete chondrogenic differentiation. Further studies are required to clarify the role of the HEY1-NCOA2 and Runx2 interaction.

In conclusion, the present study clarified that HEY1-NCOA2 expression in chondrogenic progenitors interferes with the differentiation program and induces tumorigenesis, mimicking human mesenchymal chondrosarcoma.

## Methods

### Generation of the mesenchymal chondrosarcoma model mouse.

Femoral and humeral bones of BALB/c mouse embryos (Clea Japan) were removed aseptically on 18.5 dpc, and eSZ cells were obtained by dissection using 2 mg/mL collagenase (Wako) at 37˚C for 2 hours. They were cultured in growth medium composed of IMDM (Wako) supplemented with 10% FBS and subjected immediately to retroviral infection without further purification. Retroviral stock was added into the medium containing osteochondrogenic progenitors with 10 μg/mL of polybrene (Nakalai Tesque), which was then spun at 700*g* for 1 hour. The spin infection was repeated after 24 hours. Transduced mesenchymal osteochondrogenic progenitors were mixed with Matrigel (Becton Dickinson), and 1 × 10^6^ cells were transplanted into the subcutaneous regions of BALB/c nude mice.

### Cell culture, plasmids, and human sarcoma specimens.

Mouse mesenchymal chondrosarcoma cell lines derived from the mouse mesenchymal chondrosarcoma model were cultured in IMDM supplemented with 10% FBS.

N-terminal FLAG-tagged HEY1-NCOA2 was introduced into the pMYs-IRES-GFP vector. Full-length HEY1-NCOA2 was cloned from a human mesenchymal chondrosarcoma case. pEF-Bos-Runx2 and pEF-Bos-Runx3 (wild-type, mouse) are previously described (56). pMYs-5xmyc-Neo-Runx2 was generated by ligating the PCR-amplified Runx2 sequence to the pMYs-5xmyc-Neo vector. HEY1-NCOA2 deletion mutants were produced by a KOD+ mutagenesis kit (Toyobo) with specific primers. The sequence of the mutant products was verified by Sanger sequencing.

Mesenchymal chondrosarcoma surgical specimens were obtained from adult patients at the Cancer Institute Hospital.

### Histopathology, immunohistochemistry, and immunoblotting.

Formaldehyde- or paraformaldehyde-fixed tumor tissues were embedded in paraffin, and sections were stained with H&E using standard techniques. The following primary antibodies were used: anti-FLAG M2 (Sigma-Aldrich), anti-Sox9 (Sigma-Aldrich), anti-Hey1 (Abcam), anti-Runx2 (MBL), anti-CK18 (Proteintech), anti-Galectin 3 (Proteintech), anti-Ki-67 (Abcam), and anti-NCOA2/SRC2 (Cell Signaling).

Immunoblotting was performed using whole-cell lysates. The following primary antibodies were used: Anti-FLAG M2 (Sigma-Aldrich), anti-Runx2 (MBL), anti-Runx3 (Abcam), anti–α-tubulin (Sigma-Aldrich), and anti-Cas9 (Novus, Centennial, CO).

### Coimmunoprecipitation assays.

293T cells were cotransfected with FLAG-tagged full-length or deletion mutants of HEY1-NCOA2 and with 5xmyc-tagged Runx2. Cells were lysed 48 hours after transfection in RIPA buffer or TNE buffer with protease inhibitor cocktail (Nakalai Tesque) and incubated with rabbit polyclonal anti-FLAG (Sigma-Aldrich) and anti-Runx2 (Cell Signaling) overnight at 4°C and immunoprecipitated with Dynabeads Protein G beads (Invitrogen) for 2 hours at 4°C. After washing 3 times, the precipitated proteins were eluted by boiling in Laemmli sample buffer. Proteins were separated by sodium dodecyl SDS-PAGE, blotted onto nitrocellulose membranes, and incubated with the indicated primary antibodies.

### Real-time quantitative PCR.

Total RNA was extracted using the FastGene RNA Basic Kit/Premium Kit (NIPPON Genetics) according to the manufacturer’s protocol. cDNA was synthesized from 1 μg total RNA using a reverse transcription system (Promega). Real-time qRT-PCR was performed using a 7500 Fast Real-Time PCR System (Applied Biosystems). The primers used are listed in Supplemental Table 7.

### Microarray analysis.

GeneChip analysis was conducted to determine gene expression profiles. The murine HT MG-430 PM array (Affymetrix) was hybridized with aRNA probes generated from eSZs 48 hours after transduction with pMYs-HEY1-NCOA2 or empty vector, 11 mesenchymal chondrosarcoma, 3 Ewing sarcoma, 6 synovial sarcoma, and 8 alveolar soft part sarcoma cell lines according to methods described previously (33). A total of 11 mesenchymal chondrosarcoma samples, osteochondrogenic progenitor cells, and other sarcomas were compared. Expression data were analyzed using GeneSpring (Agilent Technologies), and GSEA was performed using GSEA software (57). Gene ontology analysis was performed using IPA.

### RNA interference assays and pharmacological experiments.

shRNAs against human *HEY1* and mouse *Runx2* were lentivirally introduced into mouse mesenchymal chondrosarcoma cell lines. Knockdown efficiencies of each gene were confirmed by immunoblotting using the primary antibodies. The list of shRNAs is shown in Supplemental Table 8.

Mouse mesenchymal chondrosarcoma cells were treated with panobinostat in vitro. Cells were seeded into 96-well plates at a concentration of 5 × 10^3^ cells and were treated with drugs for 48 hours. Cell proliferation analysis was then performed using a XTT kit (Roche), and IC_50_ was calculated. DNA synthesis analysis was performed using the Click-it EdU cell proliferation kit (Thermo Fisher Scientific). FACS analysis using the BD FACSLyric system (BD Bioscience) was performed for 48 hours after treatment with 10 nM drugs to detect EdU-positive fractions. For in vivo experiments, 5 × 5 mm tumor masses were transplanted subcutaneously into BALB/c nude mice, and the mice were treated with panobinostat or adriamycin (Selleckchem). Panobinostat was intraperitoneally administered at 10 mg/kg 5 times continuously per week for 2 weeks, and adriamycin was intraperitoneally 3 mg/kg once a week for 2 weeks.

### ChIP and sequencing.

ChIP-Seq was carried out using the method previously described using a biological duplicate (58). Briefly, 5 × 10^6^ mesenchymal chondrosarcoma cells per immunoprecipitation were cross-linked with 1% formaldehyde for 10 minutes at room temperature. Chromatin was sheared in SDS lysis buffer containing 1% SDS, 10 mM EDTA, and 50 mM Tris, pH 8.0, to an average size of 400–500 bp using a Covaris S220 sonicator for 15 minutes. ChIP was carried out with 5 μg anti-FLAG (Sigma-Aldrich), anti-histone H3K27ac (Active Motif), anti-histone H3K27Me3 (Abcam), anti-histone H3K4Me3 (Abcam), anti-Runx2 (Cell Signaling), or anti-Runx3 (Cell Signaling) antibodies. The antibody-bound protein/DNA complexes were immunoprecipitated using ChIP grade protein G magnetic beads (Cell Signaling).

Immunoprecipitated DNA was then purified and subjected to secondary sonication to an average size of 150–350 bp. Libraries were prepared according to instructions accompanying the ThruPLEX DNA-Seq kit (Rubicon Genomics). The ChIP DNA was end modified and adapters were ligated. DNA was PCR amplified with Illumina primers, and Illumina-compatible indexes were added. The library fragments of approximately 300-500 bp were band-isolated from an agarose gel. The purified DNA was sequenced on an Illumina MiSeq next-generation sequencer following the manufacturer protocols.

### ChIP-Seq data analysis.

Base calls were carried out using Bowtie2-2.2.5 (http://bowtie-bio.sourceforge.net/bowtie2/index.shtml). ChIP-Seq reads were aligned to the mm9 (https://www.ncbi.nlm.nih.gov/assembly/GCF_000001635.18) or hg19 (https://www.ncbi.nlm.nih.gov/assembly/GCF_000001405.13/) genome assembly using samtools 1.2 (http://www.htslib.org). Peak calling was carried out using MACS1.4 (https://github.com/macs3-project/MACS). Peak distribution was calculated by Cistrome (http://cistrome.org/ap/root). Neighbor genes on enriched genomic regions were determined using by Nucleus (https://rias.rhelixa.com).

The genomic distributions of DNA binding peaks were visualized by NGSplot (https://anaconda.org/bioconda/r-ngsplot). DNA binding of ChIP-Seq data was visualized using IGV_2.3.80 (http://software.broadinstitute.org/software/igv). The de novo motif enrichment was performed using HOMER v 4.11.1 (http://homer.ucsd.edu/homer/motif). SEs were identified using the method previously described with the ROSE program (http://younglab.wi.mit.edu/super_enhancer_code.html). Gene ontology analysis for nearby genes was performed using the GREAT software version 4.0.4 (http://great.standford.edu/public/html).

### CRISPR/Cas9-mediated gene editing.

sgRNA sequence was designed using the Mouse CRISPR Knockout Pooled Library (GeCKO v2) (https://www.addgene.org/pooled-library/zhang-mouse-gecko-v2/) and cloned into the *Bsm*bI restriction sites of lentiCRISPR v2. gRNA target sequences are listed in Supplemental Table 7. Lentiviral supernatants were generated using 293FT transfected with Virapower Packaging Mix (Thermo Fisher) and lipofectamine (Thermo Fisher). Mesenchymal chondrosarcoma C24 cells were transduced with lentiviral supernatant supplemented with polybrene. At 24 hours after transduction, transduced cells were selected with 5 μg/mL puromycin. A single clone was isolated, and sequence was confirmed by Sanger sequencing. Runx2 and Runx3 expression was also confirmed by Western blotting.

### scRNA-Seq.

A total of 200 mg tumor tissue was dissected into small pieces and digested using a Tumor Dissociation Kit and GentleMACS Dissociator (Miltenyi Biotec). The digested tumor tissue was filtered through a 70 μm MACS SmartStrainer (Miltenyi Biotec), and the mass remaining on the filter was dissociated using Accutase (Nacalai Tesque) to obtain a single-cell suspension. The scRNA-Seq library was prepared using a BD Rhapsody Single-Cell Analysis system (BD) following the manufacturer’s instruction. Briefly, 20,000 cells were loaded onto the BD Rhapsody microwell cartridge, and cDNAs were synthesized using a BD Rhapsody Whole Transcriptome Analysis Amplification Kit. Gene expression libraries were sequenced on the Illumina NextSeq 550 platform (Illumina) with paired-end reads (read 1, 75 bp; index 1, 8 bp; read 2, 75 bp). Sequencing data were processed using BD Rhapsody Analysis pipelines on the Seven Bridges Genomics platform and converted to the gene expression count matrix.

### Single-cell data analysis.

Cell quality control and unsupervised clustering was performed using the R package Seurat (59). The data were filtered to remove cells with fewer than 400 and over 4,000 unique genes per cell and over 15% of mitochondrial expression. The filtered data were processed with Seurat’s standard pipeline using the following steps. (a) Normalized data were run using “Log Normalize” method with scale.factor of 10,000; (b) highly variable features were identified by Find Variable Features using “vst” method and 2,000 features; (c) data scaling and principal components computing were performed by Scale Data and Run PCA; (d) cell clustering was conducted by Find Neighbors function with 1–20 dimensions and Find Clusters function with a resolution of 0.5; and (e) the cells were projected onto the UMAP embedding space by Run UMAP with 1–20 dimensions. Clusters expressing immune, blood cell, vascular, and fibroblast markers were determined to distinguish nonneoplastic stromal cells from tumor clusters. To identify chondrocyte subtypes, clusters expressing stage-specific chondrocyte markers were extracted and further analyzed. For trajectory analysis, the R package Slingshot v1.8.0 was applied. The chondrocyte clusters were subsetted and the analysis was performed by slingshot (60) with the dimensionality reduction produced by UMAP and Seurat cluster assignments. Pseudotime for each cell was obtained by slingshot pseudotime.

### Identification of retroviral integration sites.

To identify integration sites of the *HEY1-NCOA2*–expressing retrovirus, inverse PCR was performed according to the method previously described (32).

### Material availability.

Plasmids generated in this study are available upon reasonable request (TN).

### Data availability.

Microarray, ChIP-Seq, and scRNA-Seq data are accessible through the NCBI Gene Expression Omnibus (GEO) database (accession GSE163291, GSE163585, and GSE198662, respectively).

### Statistics.

All data are representative of results from at least 3 independent experiments unless otherwise specified in the figure legends. The mean ± SD of individual experiments is shown. Student’s 2-tailed *t* test and 1-way ANOVA were used. *P* values of less than 0.05 were considered significant.

### Study approval.

All animal experiments described in this study were performed in strict accordance with standard ethical guidelines and were approved by the animal care committee at the Japanese Foundation for Cancer Research (approvals 10-05-9 and 0604-3-13). Mesenchymal chondrosarcoma surgical specimens were obtained from adult patients at the Cancer Institute Hospital. This study was approved by Institutional Review Board of the Japanese Foundation for Cancer Research (approval 2013-1155) and was conducted according to the tenets of the Helsinki Declaration. Informed consent was obtained from all donors.

## Author contributions

MT provided investigation, formal analysis, data curation, visualization, and validation; acquired funding; and wrote the original draft. MH provided investigation and validation. YT provided investigation and formal analysis. KK provided formal analysis and data curation. YY provided investigation. KY provided resources. MO provided resources and supervision. RM provided data curation and formal analysis and wrote the original draft. TN conceptualized the study; provided investigation, project administration, and supervision; acquired funding; and wrote the original draft and edited the manuscript.

## Supplementary Material

Supplemental data

Supplemental table 1

Supplemental table 2

Supplemental table 3

Supplemental table 4

Supplemental table 5

Supplemental table 6

Supplemental table 7

Supplemental table 8

## Version 1 05/22/2023

Electronic publication
