# Supplementary material for: HEY1-NCOA2 expression modulates chondrogenic differentiation and induces mesenchymal chondrosarcoma in mice
Source: JCI Insight. 2023 May 22;8(10):e160279. doi: 10.1172/jci.insight.160279 (PMC10322689; doi:10.1172/jci.insight.160279)
Supplement: Supplemental data [file jciinsight-8-160279-s171.pdf]

Supplemental Materials

HEY1-NCOA2 expression modulates chondrogenic differentiation and induces mesenchymal chondrosarcoma in mice

Miwa Tanaka, Mizuki Homme, Yasuyo Teramura, Kohei Kumegawa, Yukari Yamazaki, Kyoko Yamashita, Motomi Osato, Reo Maruyama, Takuro Nakamura

7 Supplemental Figures with legends

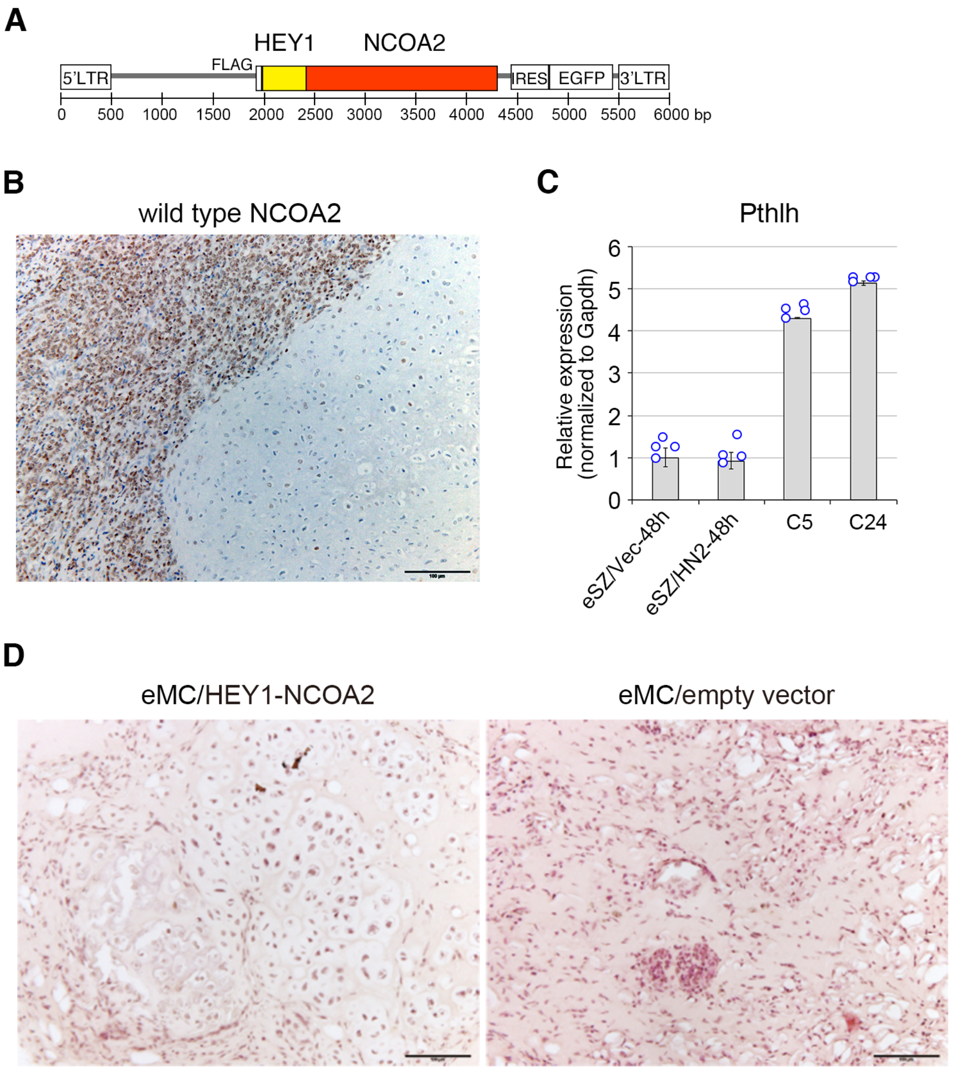

Supplemental Figure 1. Generation of the mouse model for human mesenchymal chondrosarcoma. **(A)** The structure of FLAG-HEY1-NCOA2 in the pMYs retroviral vector. **(B)** Expression of HEY1-NCOA2 in human mesenchymal sarcoma detected with the anti-NCOA2 C-terminal antibody. Scale bar, 100 µm. **(C)** qRT-PCR showing expression of *Pthlh* in eSZ and mesenchymal chondrosarcoma cells. **(D)** Development of mature cartilage tissue by HEY1-NCOA2 expression in mouse embryonic mesenchymal cells (eMCs) (left). No cartilage development in eMC introduced with empty vector (right). Scale bar, 100 µm.

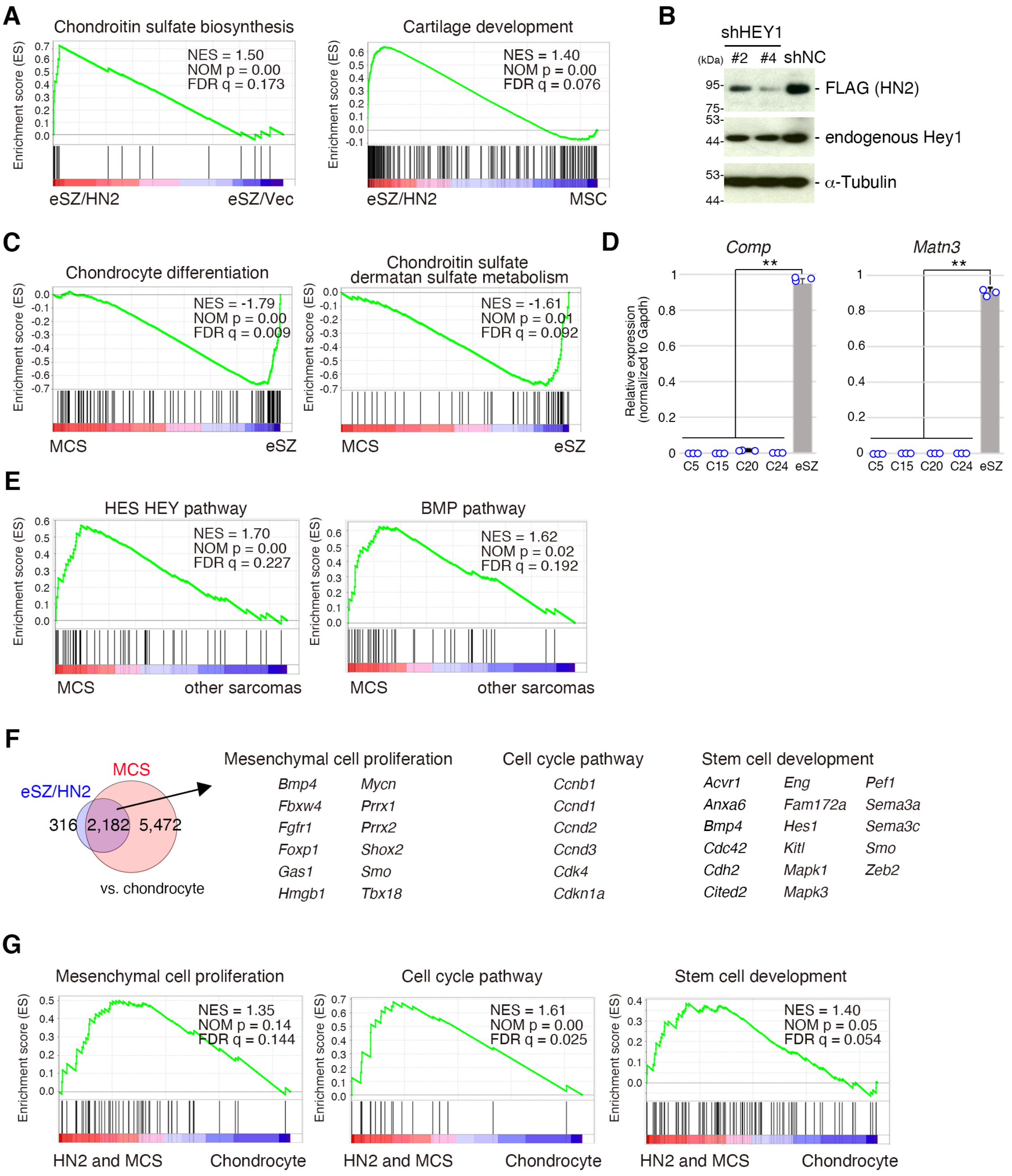

Supplemental Figure 2. The gene expression profile of murine mesenchymal chondrosarcoma. **(A)** GSEA shows correlation between the chondroitin sulfate biosynthesis pathway and genes involved in eSZ cells expressing *HEY1-NCOA2* (left). Enrichment of the cartilage development pathway was observed when gene expression of eSZ cells with *HEY1-NCOA2* was compared with MSC-rich embryonic limb mesenchymal cells (MSC) (right). **(B)** Western blotting showing the knockdown effect of HEY1-NCOA2 (FLAG) by human *HEY1*-specific short hairpin RNA #2 and #4. Note equal expression levels of endogenous Hey1. **(C)** GSEA shows inverse correlation

between the chondrocyte differentiation (left) and chondroitin sulfate dermatan sulfate metabolism (right), and genes involved in mesenchymal chondrosarcoma (MCS) compared with eSZ cells. **(D)** Downregulation of *Comp* and *Matn3* in mesenchymal chondrosarcoma cells. Statistical analyses was performed by one-way ANOVA. ** $P < 0.01$. **(E)** GSEA shows correlation between the HES/HEY and BMP pathways, and genes involved in mesenchymal chondrosarcoma (MCS) compared with other sarcomas. **(F)** Venn diagram showing upregulated genes in *HEY1-NCOA2*-expressing eSZ cells versus chondrocytes, or mesenchymal chondrosarcoma versus chondrocytes. The expression data for chondrocytes used were available in the NCBI Gene Expression Omnibus (GEO) database (http://www.nci.nlm.nih.gov/geo) under the accession number GSE27177. **(G)** Genetic pathways such as mesenchymal development, cell cycle, and stem cell development were enriched in eSZ cells with HEY1-NCOA2 and mesenchymal chondrosarcoma cells.

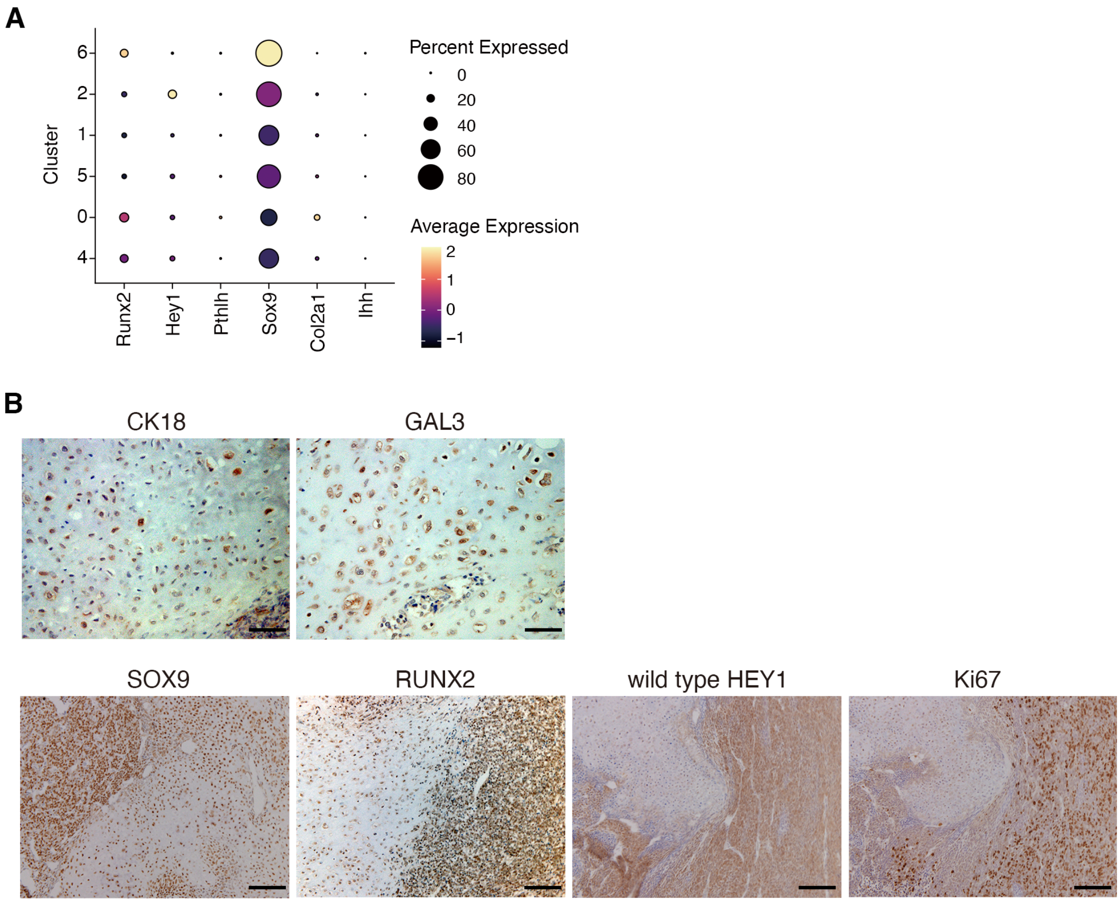

Supplemental Figure 3. Chondrocyte marker gene expression in mesenchymal chondrosarcoma. (A) Dot plot analysis showing distributions of *Runx2, Hey1, Pthlh, Sox9, Col2a1,* and *Ihh* expression in six tumor cluster of mouse mesenchymal chondrosarcoma. **(B)** Immunohistochemical analysis demonstrates CK18 and GAL3 expression in the mature cartilage component in human mesenchymal chondrosarcoma (top). Distinct expression patterns with partial overlapping of Sox9, Runx2, endogenous Hey1 and Ki67 (bottom). Scale bar; 50 µM (top) and 100 µm (bottom).

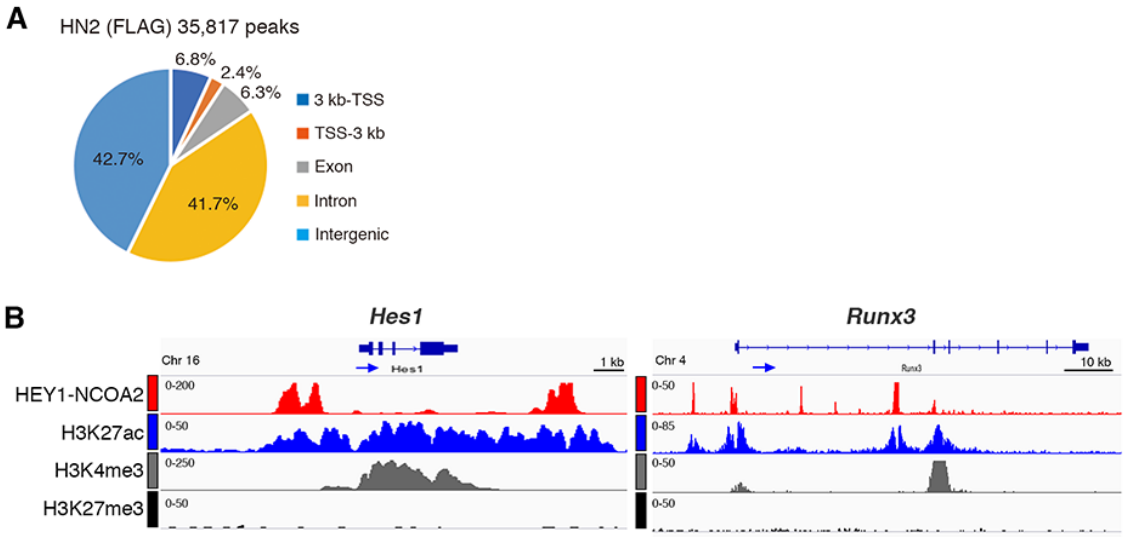

Supplemental Figure 4. HEY1-NCOA2 binding sites in mesenchymal chondrosarcoma. (A) Global distribution of HEY1-NCOA2 binding peaks in the mouse mesenchymal chondrosarcoma cell C5. **(B)** ChIP-seq occupancy profiles for *Hes1* and *Runx3* loci. Arrows indicate transcriptional orientation.

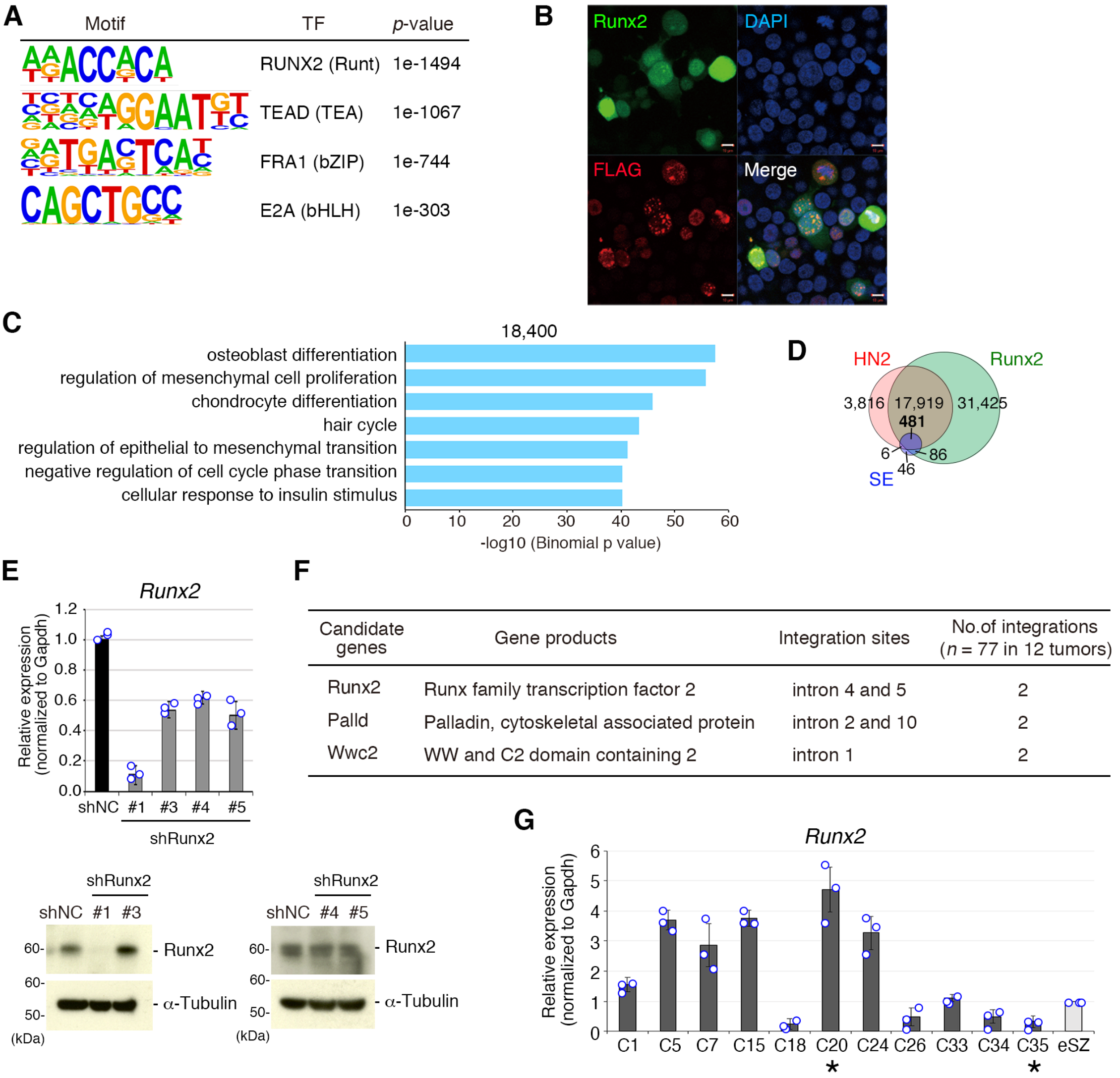

Supplemental Figure 5. Association between HEY1-NCOA2 and Runx2 in DNA binding. (A) HOMER motif analysis showing enrichment of the RUNX2 motif in HEY1-NCOA2 binding peaks detected in mouse mesenchymal chondrosarcoma cell C5. *p* values were calculated using Fischer's exact test. **(B)** Immunofluorescent assessment of the expression of FLAG-tagged HEY1-NCOA2 and exogenous Runx2 in HEK293T cells. Scale bar; 10 µm. **(C)** Enrichment of gene pathways for 18400 HEY1-NCOA2 and Runx2 overlapping peaks indicated in Figure 5G. *p* value was calculated using a binominal test. **(D)** Venn diagram showing overlapping among SEs, HEY1-NCOA2 (HN2), and Runx2 binding peaks. **(E)** shRNA-mediated knockdown of *Runx2*. qRT-PCR (left) and western blotting (right) showing knockdown effects of 4 different shRNAs. #1 was used in subsequent experiments. **(F)** Common retroviral integration sites in 12 mouse mesenchymal chondrosarcomas. **(G)** Expression of *Runx2* in mouse mesenchymal chondrosarcomas. Asterisks indicate the tumor with retroviral integrations at *Runx2* loci.

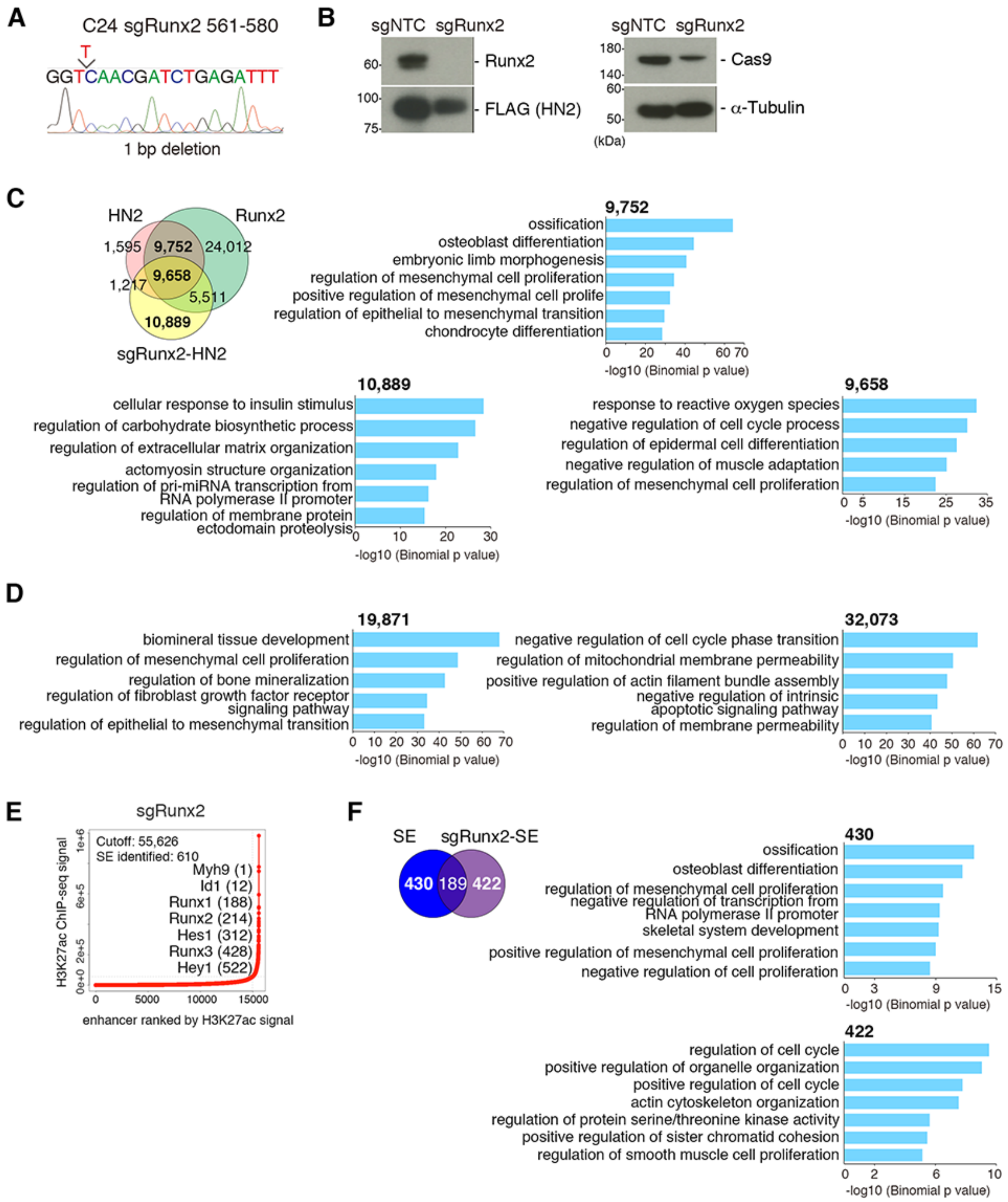

Supplemental Figure 6. Transcriptional regulation by HEY1-NCOA2 and Runx2 collaboration. **(A)** CRISPR/Cas9-mediated knockout of *Runx2* in the mouse mesenchymal chondrosarcoma cell C24. Sequence analysis showing 1 bp deletion within the *Runx2* coding sequence. **(B)** Western blotting shows disappearance of Runx2 (left) and expression of Cas9 (right). **(C)** Venn diagram showing the overlapped distribution of Runx2 and HEY1-NCOA2 (HN2) with or without *Runx2* knockout (top, left). Enrichment of gene pathways for 9752 peaks specific to HEY1-NCOA2 and Runx2 overlapping at

the *Runx2*-positive state (top, right), 10889 peaks specific to HEY1-NCOA2 at the *Runx2*-negative condition (bottom, left), and 9658 all 3 overlapping peaks (bottom, right). (D) **(E)** Enhancers were ranked by increasing H3K27ac ChIP-seq signals in mesenchymal chondrosarcoma cells with *Runx2* knockout. Using the ROSE algorithm, 610 enhancers were defined as super-enhancers. **(F)** Venn diagram showing the overlapped distribution of SEs with or without *Runx2* knockout (top, left). Enrichment of gene pathways for 430 SEs in the *Runx2*-positive state (top, right) and 422 SEs in the *Runx2*-null state (bottom, right). *p* values in **C, D**, and **F** were calculated using a binominal test.

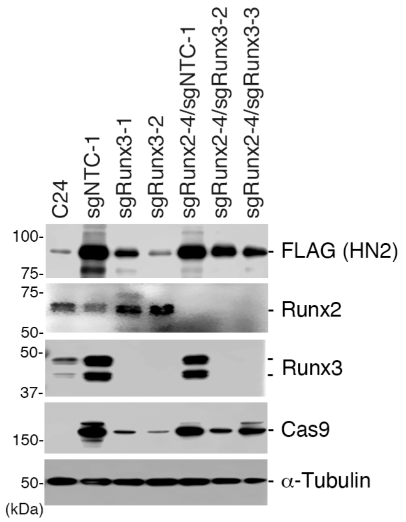

Supplemental Figure 7. CRISPR/Cas9-mediated knockout of *Runx3*. Immunoblotting shows deletion of Runx3 and/or Runx2 in C24 mesenchymal chondrosarcoma cells.
